# Integrative metabolomics highlights gut microbiota metabolites as novel NAFLD-related candidate biomarkers in children

**DOI:** 10.1128/spectrum.05230-22

**Published:** 2024-03-06

**Authors:** Jiayou Luo, Miyang Luo, Atipatsa C. Kaminga, Jia Wei, Wen Dai, Yunlong Peng, Kunyan Zhao, Yamei Duan, Xiang Xiao, SiSi Ouyang, Zhenzhen Yao, Yixu Liu, Xiongfeng Pan

**Affiliations:** 1 Pediatrics Research Institute of Hunan Province, Hunan Children’s Hospital, Changsha, China; 2 Department of Maternal and Child Health, Xiangya School of Public Health, Central South University, Changsha, China; 3 Department of Epidemiology and Health Statistics, Xiangya School of Public Health, Central South University, Changsha, China; 4 Saw Swee Hock School of Public Health, National University of Singapore, Singapore, Singapore; 5 Department of Mathematics and Statistics, Mzuzu University, Mzuzu, Malawi; 6 Department of Epidemiology and Health Statistics, Medical College of Soochow University, Suzhou, China; 7 School of Public Health, University of South China, Hengyang, China; Tainan Hospital, Ministry of Health and Welfare, Tainan, Taiwan

**Keywords:** metabolomic, gut microbiota, non-alcoholic fatty liver disease, children, biomarker

## Abstract

Altered gut microbiota and metabolites are important for non-alcoholic fatty liver disease (NAFLD) in children. We aimed to comprehensively examine the effects of gut metabolites on NAFLD progression. We performed integrative metabolomics (untargeted discovery and targeted validation) analysis of non-alcoholic fatty liver (NAFL), non-alcoholic steatohepatitis (NASH), and obesity in children. Fecal samples were collected from 75 subjects in the discovery cohort (25 NAFL, 25 NASH, and 25 obese control children) and 145 subjects in an independent validation cohort (53 NAFL, 39 NASH, and 53 obese control children). Among 2,491 metabolites, untargeted metabolomics revealed a complete NAFLD metabolic map containing 318 increased and 123 decreased metabolites. Then, machine learning selected 65 important metabolites that can distinguish the severity of the NAFLD. Furthermore, precision-targeted metabolomics selected 5 novel gut metabolites from 20 typical metabolites. The functionality of candidate metabolites was validated in hepatocyte cell lines. In the end, this study annotated two novel elevated pathogenic metabolites (dodecanoic acid and creatinine) and a relationship between depleted protective gut microbiota (*Butyricicoccus* and *Alistipes*), increased inflammation (IL-1β), lipid metabolism (TG), and liver function (ALT and AST). This study demonstrates the role of novel gut metabolites (dodecanoic acid and creatinine), as the fatty acid metabolism regulator contributing to NAFLD development through its influence on inflammation and liver function.

**IMPORTANCE:**

Altered gut microbiota and metabolites are a major cause of non-alcoholic fatty liver disease (NAFLD) in children. This study demonstrated a complete gut metabolic map of children with NAFLD, containing 318 increased and 123 decreased metabolites by untargeted metabolomic. Multiple validation approaches (machine learning and targeted metabolomic) selected five novel gut metabolites for targeted metabolomics, which can distinguish NAFLD status and severity. The gut microbiota (*Butyricicoccus* and *Alistipes*) and metabolites (creatinine and dodecanoic acid) were novel biomarkers associated with impaired liver function and inflammation and validated by experiments of hepatocyte cell lines. The data provide a better understanding of the importance of gut microbiota and metabolite alterations in NAFLD, which implies that the altered gut microbiota and metabolites may represent a potential target to prevent NAFLD development.

## INTRODUCTION

Non-alcoholic fatty liver disease (NAFLD) is now considered as the most prevalent chronic liver disease in children worldwide, with approximately 52.49% of obese children being estimated to have the NAFLD (1). Moreover, NAFLD is a broad spectrum of chronic liver diseases ranging from non-alcoholic fatty liver simple steatosis (NAFL) to non-alcoholic steatohepatitis (NASH), fibrosis, cirrhosis, and ultimately hepatocellular carcinoma. Medical requirements for NAFLD incur huge economic burdens (2). Obesity is a risk factor for NAFLD, but not all obese children develop NAFLD (3). Noteworthy, multiple factors may play a role in the development of NAFLD, but its etiology, pathogenesis, and mechanistic insights remain largely unknown (4). Recent studies suggest that the gut-liver axis may be one of the most promising breakthroughs in the pathogenesis of NAFLD (5, 6).

Gut microbiota has been reported to play an important role in the development of NAFLD. For example, for adults, *Bacteroides*, *Streptococcus*, and *Ruminococcus* were significantly elevated in NAFLD, whereas *Akkermansia*, *Alkaliphilus*, *Prevotella*, and *Bacteroidetes* were decreased in the controls (7–9). Also, for children, *Akkermansia*, *Oscillibacter*, *Prevotella*, and *Enterococcus* were significantly elevated in NAFLD, whereas *Bacteroides*, *Bifidobacterium*, *Bifidobacterium*, and *Blautia* were decreased in the controls (5, 10, 11). Thus, associations of NAFLD with *Akkermansia* (5, 9), *Bacteroides* (8, 11), and *Prevotella* (7, 10, 11) had a different pattern between adults and children.

Moreover, several studies investigated metabolites of NAFLD. They found that, for adults, bile acids, sphingolipid, betaine, trimethylamine oxide, and SCFAs were significantly elevated in NAFLD, whereas glutamic acid, serine, and aspartic acid were decreased in the controls (12–14). Also, for children, phenol, 2-butanone, 4-methyl-2-pentanone, and aspartate were significantly elevated in NAFLD, whereas betaine, 1-pentanol, formate, and SCFAs were decreased in the controls (15–17). Therefore, while there is a general agreement that the composition of gut metabolites is altered in NAFLD, associations of betaine (13, 15) and SCFAs (14, 16, 17) with NAFLD had inconsistent patterns between adults and children.

However, the foregoing studies were often limited to a single metabolite or a class of metabolites and may have missed many important metabolites (18, 19). Thus, more extensive metabolomic explorations were needed, such as amino acids, benzenoids, bile acids, fatty acids, organosulfonic acids, phenols, and purine nucleotides. In addition, the lack of repeated validation in population and *in vitro* cell experiments may lead to false-positive results. Also, whether metabolites are related to impaired glycolipid metabolism, liver function, gut microbiota, and inflammation has not been reported. Our previous study showed that the gut microbiota of children with NAFLD was substantially changed and associated with the metabolic pathway of functional enrichment (6). This study hypothesized that gut microbiota-derived metabolites may play a key role in regulating the metabolic disorder in children with NAFLD. These changes in gut microbiota and metabolites may provide insights into the gut-liver axis and hence assist in evaluating potential etiologies and novel therapeutic targets in children with NAFLD.

Therefore, understanding changes in gut metabolites and identifying novel molecular targets may help shed more light in the therapeutical developments for NAFLD in children. In the current study, we applied untargeted discovery and multiple validation, such as targeted validation metabolomics, and *in vitro* cell experiment verification technologies, to analyze the gut metabolome changes in NAFLD children. The aim of this study was to reveal novel gut microbiota and metabolites in NAFLD children that were associated with disease severity and evolution, including liver function, glycolipid metabolism disorder, and inflammation.

## RESULTS

### Patient characteristics

**Fig 1:**
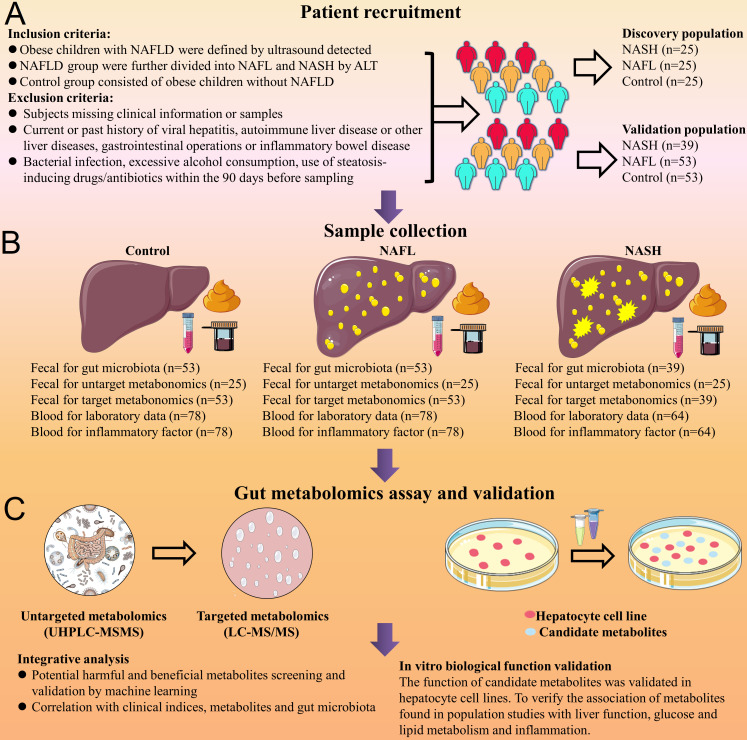
Flow chart showing the multidisciplinary approaches used for this study. (**A**) Patient recruitment, inclusion, and exclusion criteria. (**B**) Various clinical samples were obtained from NAFLD patients and controls. (**C**) Integrative analysis for potential harmful and beneficial gut metabolites based on Tax4Fun function prediction and Kyoto Encyclopedia of Genes and Genomes (KEGG) pathway enrichment analysis. The targeted metabolomics (LC-MS/MS) and untargeted metabolomics (UHPLC-MS/MS) offer a plethora of information on metabolites, the gut microbiota. NAFL, non-alcoholic fatty liver; NASH, non-alcoholic steatohepatitis; LC-MS/MS, liquid chromatography coupled to tandem mass spectrometry; UHPLC-MS/MS, ultra-high-performance liquid chromatography-tandem mass spectrometry.

We prospectively recruited an independent discovery population of children with NAFLD alongside their obese children controls and a validation population of children affected by NAFLD alongside their obese children controls. The recruitment of subjects used a strict inclusion and exclusion criteria to avoid effects on the gut microbiome and metabolites (Fig. 1A). In the discovery population, untargeted metabolomics [ultra-high-performance liquid chromatography-tandem mass spectrometry (UHPLC-MS/MS)] profiling was performed on 75 samples from 25 NAFL patients, 25 NASH patients, and 25 obese controls. In the validation population, targeted metabolomics [liquid chromatography coupled to tandem mass spectrometry (LC-MS/MS)] profiling was performed on 145 samples from 53 NAFL patients, 39 NASH patients, and 53 obese controls (Fig. 1B and C). The clinical and standard laboratory data of the different groups are shown in the Supplementary material in Appendix 1. None of the children included in this study had a history of alcohol intake. Different groups of inflammatory factors in the discovery population and validation population are shown in the Supplementary materials in Appendices 2 and 3, respectively. There was no significant difference in the dietary data of different groups for both the discovery population and validation population as shown in the Supplementary materials in Appendices 4 and 5, respectively.

### Overall untargeted metabolomics profiling and annotated metabolites

Pearson correlation of quality control (QC) was high (*R*^2^ > 0.9), indicating that the data quality of the untargeted metabolomics results was stable and accurate (Supplementary material, Appendix 6). Untargeted metabolomics revealed a complete metabolic map. A total of 218 metabolites in the positive mode and 127 metabolites in the negative mode were annotated at level 3. A total of 1,381 metabolites in the positive mode and 765 metabolites in the negative mode were annotated at level 2. Human Metabolome Database (HMDB) annotation analysis showed that the metabolites mainly included the following categories: lipids and lipid-like molecules, organic acids and derivatives, organoheterocyclic compounds, and benzenoids (Supplementary material, Appendix 7). Moreover, LIPID MAPS annotation analysis showed that the metabolites mainly included the following categories: fatty acids and conjugates, flavonoids, eicosanoids, glycerophosphoethanolamines, glycerophosphocholines, and steroids (Supplementary material, Appendix 8).

### Metabolic alterations are annotated in NAFLD patients compared with controls

**Fig 2:**
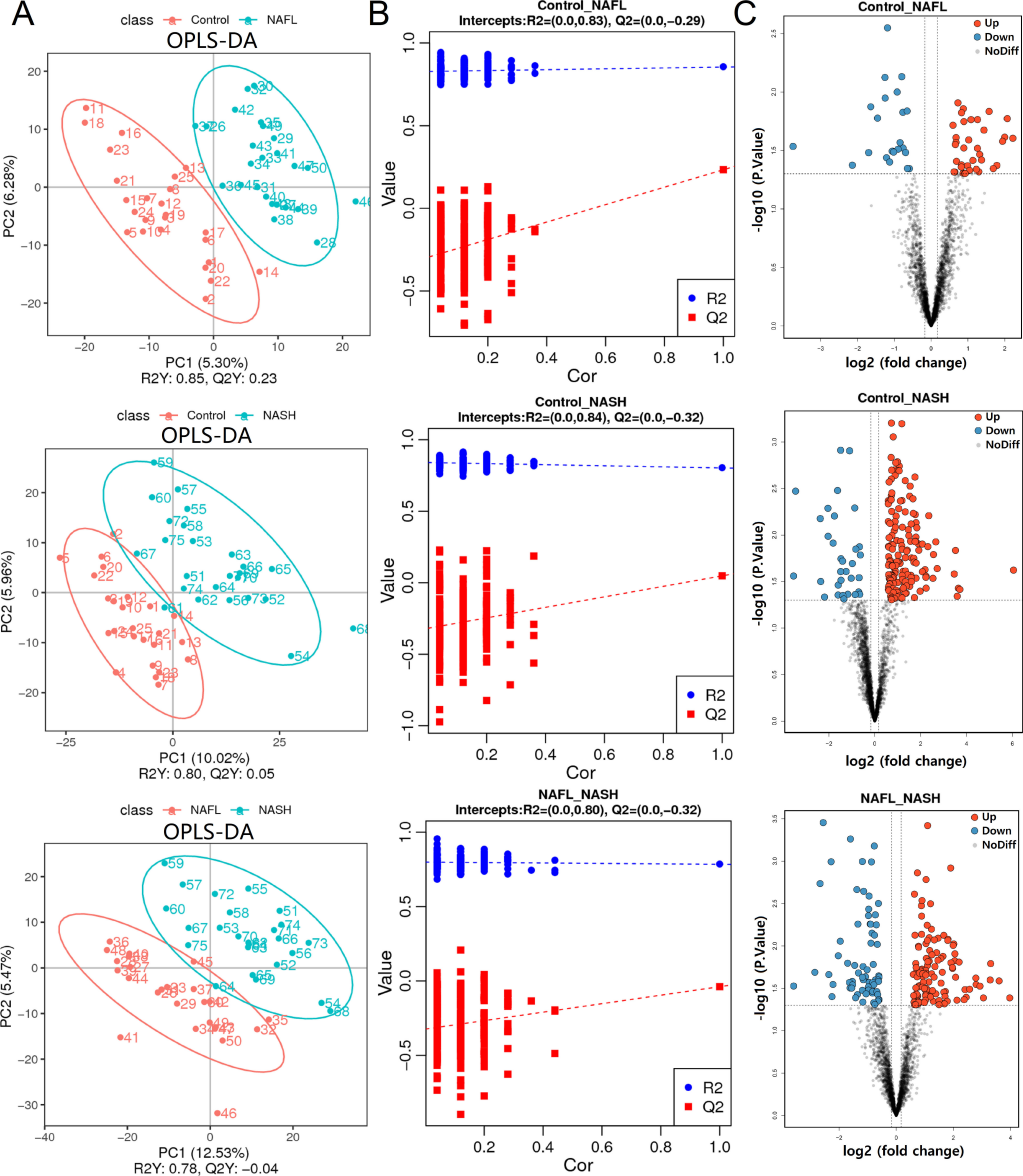
Screening of differential metabolites (OPLS-DA score scatter plot and volcano plot). (**A**) OPLS-DA score plots for differential metabolites. The X axis and Y axis represent contributions of persons to the first two principal components (PC1 and PC2). (**B**) Cross-validation plot with a permutation test repeated 200 times. The intercepts of *R*^2^ and *Q*^2^ suggest that the OPLS-DA model is not overfitting. (**C**) Volcano plots showing the results of pairwise comparisons of metabolites in each case sample’s group relative to controls. The abscissa represents the variation of multiple metabolites in different groups (log_2_ Fold Change), and the ordinate represents the significance level (−log_10_ *P* value). The vertical dashed lines indicate the threshold for the abundance difference. The horizontal dashed line indicates the *P* = 0.05 threshold. Each point in the graph represents metabolites, the upregulated metabolite as a red dot, and the downregulated metabolite as a green dot. Between-group comparisons were performed using empirical Bayes hierarchical model and Kruskal-Wallis rank sum test. OPLS-DA, orthogonal partial least squares discrimination analysis; C, control group; NAFL, non-alcoholic fatty liver; NASH, non-alcoholic steatohepatitis.

Based on the results of principal components analysis (PCA) in unsupervised mode, there was no difference between different groups (Supplementary material, Appendix 9). Therefore, we further used the method of [orthogonal partial least squares discrimination analysis (OPLS-DA)] in supervised mode to reduce the dimension of the data. Distinct clusters of metabolites in NAFLD patients compared with the controls were demonstrated in stool samples (*P* < 0.05) by OPLS-DA score plots (Fig. 2A). Intercepts of goodness-of-fit (*R*^2^) and goodness-of-prediction (*Q*^2^) models containing metabolomics information were performed by fitting an OPLS-DA, and its ability to correctly classify new samples during a sevenfold cross-validation was tested with a permutation test repeated 200 times. The intercepts of *R*^2^ and *Q*^2^ indicated that the OPLS-DA model was stable and not overfitting (Fig. 2B). Unlike in the control children, the volcano plots showed that children with NAFL had 36 significantly increased gut metabolites and 23 decreased gut metabolites and children with NASH had 160 significantly increased gut metabolites and 32 decreased gut metabolites. Also, compared with NAFL children, children with NASH had 122 significantly increased gut metabolites and 68 decreased gut metabolites [variable importance in projection (VIP) > 1.0, fold change (FC) > 1.500 or FC < 0.667, *P* < 0.05; Fig. 2C].

**Fig 3:**
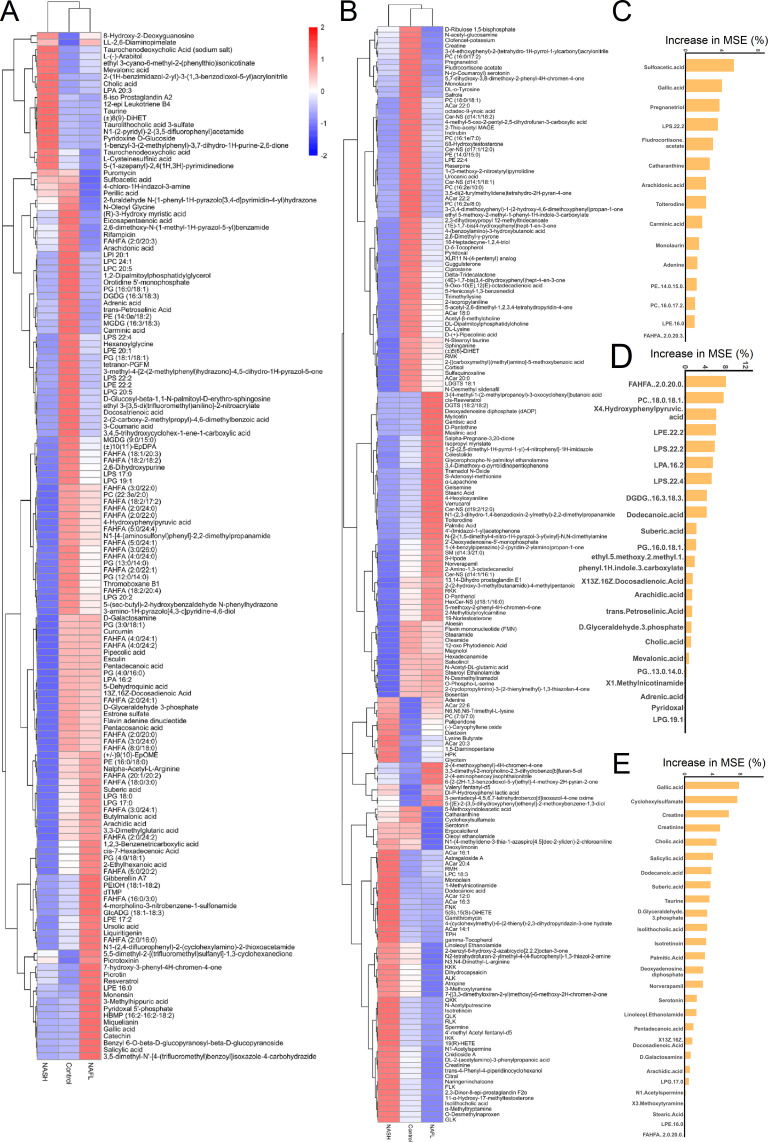
A comprehensive metabolic map and random forest analysis ranked between the different-groups heatmap of separation of NAFLD and control group by metabolomic features. NAFL, non-alcoholic fatty liver; NASH, non-alcoholic steatohepatitis. (**A**) Metabolites in the negative-mode metabolic map. (**B**) Metabolites in the positive-mode metabolic map. Separation of NAFLD and control group by machine learning of metabolomic features. (**C**) C vs NAFL group base on untargeted metabolomics. (**D**) C vs NASH group base on untargeted metabolomics. (**E**) NAFL vs NASH group base on untargeted metabolomics. Metabolites prioritized by random forest analysis ranked by the increase in MSE. C, control group; NAFL, non-alcoholic fatty liver; NASH, non-alcoholic steatohepatitis; MSE, mean decrease accuracy.

**Fig 4:**
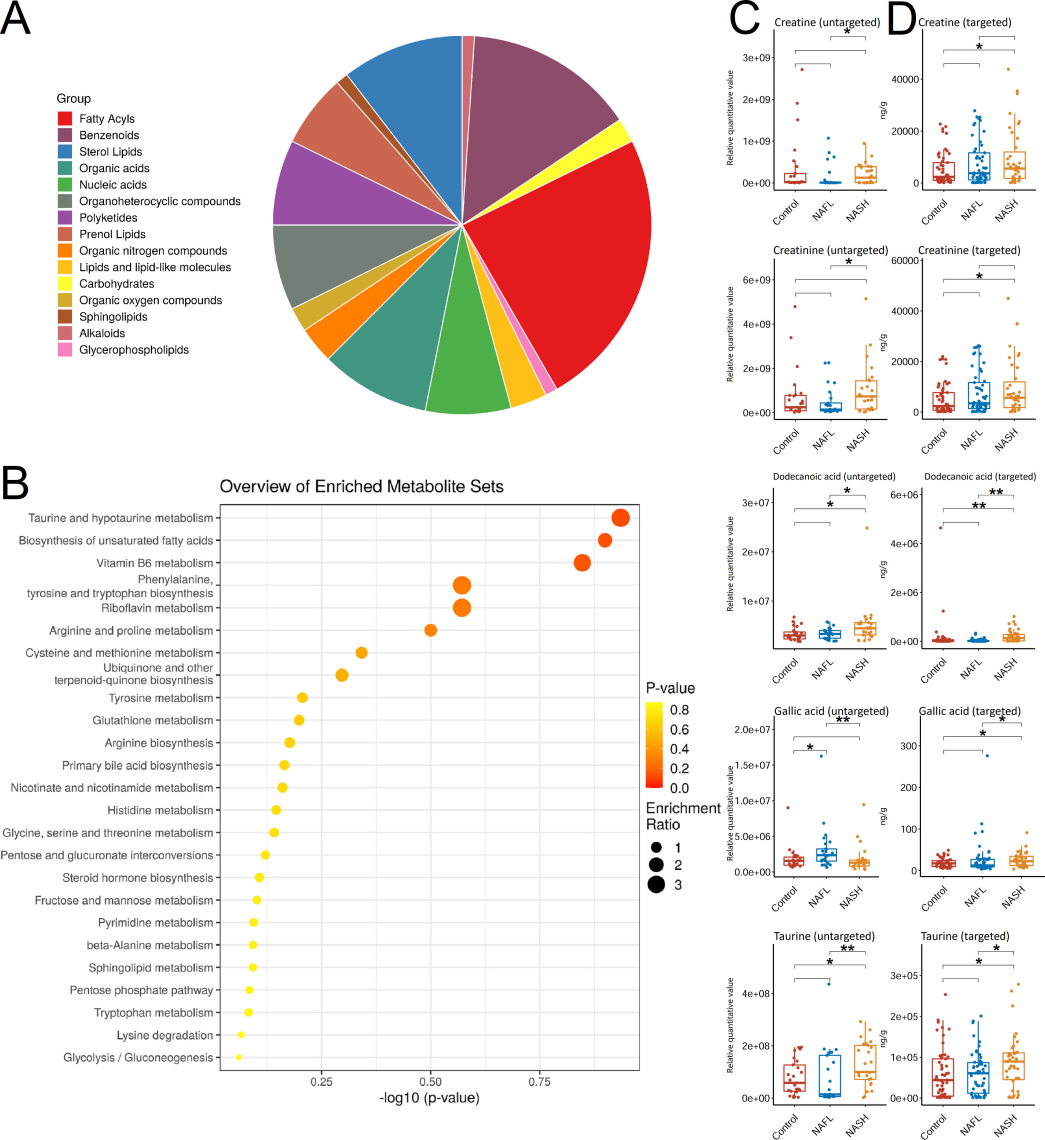
Metabolites discriminate disease severity. Chemical class metabolite sets plots (**A**) show the chemical class metabolite of metabolites and pathway enrichment plots (**B**) show the pathway enrichment analysis by KEGG for metabolites. The pathway enrichment analysis result takes KEGG pathway as the unit, and the hypergeometric test is applied to find the pathway that is enriched in the differential metabolites against the background of all annotated metabolites. Pathway enrichment analysis can determine the most important metabolic pathways and signal transduction pathways involved in the differential metabolites. Scatter plots (**C**) show the relative abundance of the fecal levels of creatine, creatinine, dodecanoic acid, gallic acid, and taurine in the control, NAFL, and NASH groups in untargeted metabolomics. Scatter plots (**D**) show the absolute concentration (ng/g) of the fecal levels of creatine, creatinine, dodecanoic acid, gallic acid, and taurine in the control, NAFL, and NASH groups in targeted metabolomics. A *P* value < 0.05 was considered statistically significant. NAFL, non-alcoholic fatty liver; NASH, non-alcoholic steatohepatitis; MSE, mean decrease accuracy; *P value < 0.05; **P value < 0.01.

Heatmap of metabolites in untargeted metabolomics with ESI− and ESI + models are presented in Fig. 3A and B. An average decrease in accuracy by random forest (RF) analysis for the top 15 metabolites (NAFL patients vs controls), 23 metabolites (NASH patients vs controls), and 27 metabolites (NAFL patients vs NASH) is presented in Fig. 3C through E, respectively. In order to understand the classification and characteristics of these selected significant metabolites in RF analysis, we conducted a classification annotation analysis and annotated the significant categories: fatty acyls, benzenoids, sterol lipids, organic acids, and polyketides (Fig. 4A). Moreover, KEGG functional characteristics and pathway enrichment analysis showed that the selected significant metabolites mainly included the following categories: taurine and hypotaurine metabolism, biosynthesis of unsaturated fatty acids, vitamin B6 metabolism, phenylalanine, tyrosine and tryptophan biosynthesis, and riboflavin metabolism (Fig. 4B).

### Analysis of selected gut metabolites by targeted LC-MS/MS

We further selected 20 typical metabolites for targeted metabolomics validation. Fecal levels of selected metabolites were determined by LC-MS/MS. These selected metabolites are detailed in the Supplementary material in Appendix 10. A total of five gut metabolites were found to have significant differences between the discovery and validation population. Scatter plots showed the relative abundance of the selected significant untargeted metabolites in NAFLD children and obese controls (Fig. 4C). Among them, the elevated metabolites of creatine, creatinine, dodecanoic acid, and taurine were significantly higher in NASH children compared with NAFL/obese controls. However, the relative abundance of gallic acid was significantly decreased in NASH patients. The comparison of absolute concentrations of these targeted metabolites in feces showed the same trend in targeted metabolomics (Fig. 4D).

### 16S rRNA sequencing of gut microbiota

**Fig 5:**
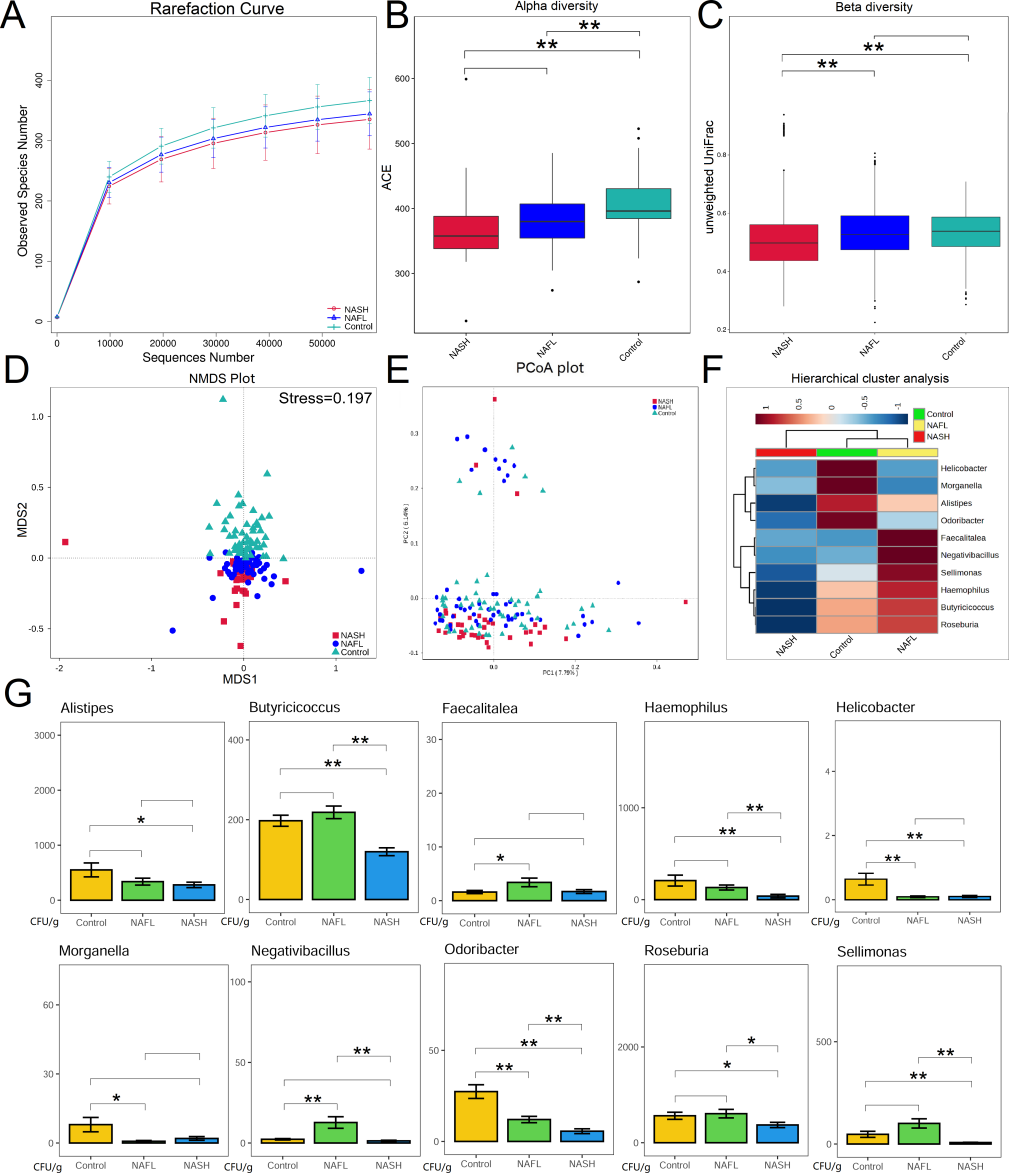
Taxonomic composition of the gut microbiota. The rarefaction curve (**A**) of the gut microbiota in different groups. Alpha (**B**) and beta (**C**) diversity indices in different groups. In the analysis of differences between groups in the alpha diversity index (Wilcoxon test based on ACE index), the higher the ACE index, the higher the species diversity. In the analysis of differences between groups in the beta diversity index (Wilcoxon test based on unweighted UniFrac), the higher the sample similarity index within the group, the higher the level of difference between the different groups. NMDS analysis in different groups (**D**). Unweighted UniFrac PCoA plot (**E**): PC1 represents one principal component, PC2 represent other principal components, and percentage represents the contribution of principal components to sample differences. Each point in the figure represents an individual, and samples in the same group are represented by the same color. Hierarchical cluster analysis (**F**) of differential gut microbiota between NAFL, NASH, and control. Red color represents the high relative abundance of gut microbiota in each group, and green color represents the low relative abundance of gut microbiota in each group. Bar chart (**G**) shows the abundance of different genus in different groups. NAFL, non-alcoholic fatty liver; NASH, non-alcoholic steatohepatitis. *P value < 0.05; **P value < 0.01.

A total of 3,362 operational taxonomic units (OTUs) were identified by paired-end read merging and error correction of 16S rRNA sequencing, including 22 phylum, 41 class, 92 order, 151 family, and 318 genus in the validation population. The rarefaction curve in Fig. 5A of this study shows that the curve tends to be flat as the number of randomly selected sequencers increases, suggesting that the amount of sequenced data is gradually reasonable and more data will only produce a small number of new species (OTUs). The alpha diversity indices of the NASH group (*P* = 0.005) and the NAFL group (*P* < 0.001) were significantly lower than those of the control groups (Fig. 5B). There was no significant difference in alpha diversity between the NAFL group and the NASH group (*P* = 0.051). The beta diversity index (Fig. 5C) of the NASH group was significantly lower than that of the NAFL group (*P* < 0.001) and control group (*P* < 0.001). There was no significant difference in beta diversity between the NAFL group and the control group (*P* = 0.097). NMDS analysis (Fig. 5D) showed that the difference in gut microbiota community composition between the NASH group and the NAFL group was small, while the difference in gut microbiota community composition between control group and case group was large (stress = 0.197). We found 10 gut microbiota genera that were significantly different in NAFL or NASH unlike in the controls. Unweighted UniFrac PCoA analysis showed that the distributions of the NASH and control groups were different in the PCoA plot (Fig. 5E). Hierarchical cluster analysis of differential gut microbiota between NAFL, NASH, and control children is shown in Fig. 5F. The bar chart of gut microbiota genera of different groups is shown in Fig. 5G. A total of 10 genera were found to have significant differences in the validation population (*Alistipes*, *Butyricicoccus*, *Faecalitalea*, *Haemophilus*, *Helicobacter*, *Morganella*, *Negativibacillus*, *Odoribacter*, *Roseburia*, and *Sellimonas*).

### The correlation between selected metabolites with NAFLD indices

**Fig 6:**
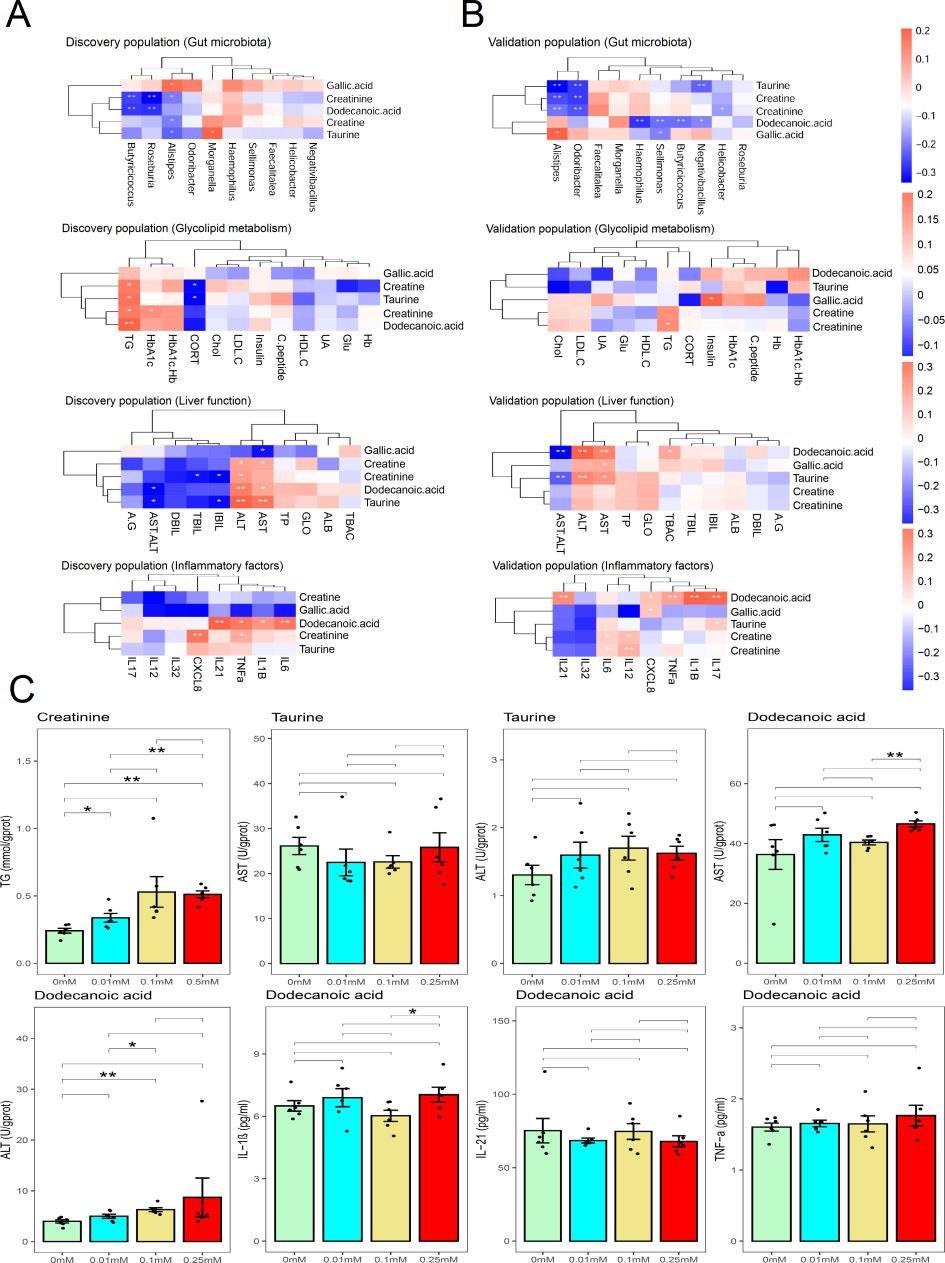
The correlation between selected metabolites with glycolipid metabolism, liver function, and inflammatory factors. A heatmap of discovery population (**A**) and validation population (**B**) shows the correlation between selected metabolites with glycolipid metabolism, liver function, and inflammatory factor indices. Red color represents positive correlation, and blue color represents negative correlation. Bar chart (**C**) shows the glycolipid metabolism, liver function, and inflammatory factor levels of THLE-3 cells affected by interested metabolites. *P value < 0.05; **P value < 0.01.

The selected significant metabolites were compared with gut microbiota and biochemical parameters in the discovery population. In this regard, a comparison with the biochemical parameters of glycolipid metabolism found that creatinine (*r* = 0.28, *P* = 0.016) had a positive correlation with the level of TG (Table 1; Fig. 6A). Also, when compared with the gut microbiota (genus), it was found that *Alistipes* had a positive correlation with the level of gallic acid (*r* = 0.26, *P* = 0.025), but a negative correlation with the level of creatine (*r* = −0.23, *P* = 0.047), creatinine (*r* = −0.23, *P* = 0.044), and taurine (*r* = −0.27, *P* = 0.021) (Table 1; Fig. 6A). Furthermore, *Butyricicoccus* had a negative correlation with the level of dodecanoic acid (*r* = −0.32, *P* = 0.005) (Table 1; Fig. 6A). Additionally, a comparison with the biochemical parameters of inflammatory factors yielded a positive correlation between dodecanoic acid and the levels of IL-1β (*r* = 0.26, *P* = 0.026), IL-21 (*r* = 0.32, *P* = 0.005), and TNF-α (*r* = 0.30, *P* = 0.010) (Table 1; Fig. 6A). Likewise, when compared with the biochemical parameters of liver function, it was observed that dodecanoic acid (*r* = 0.32, *P* = 0.005 and *r* = 0.23, *P* = 0.045) and taurine (*r* = 0.37, *P* = 0.001 and *r* = 0.33, *P* = 0.003) had a positive correlation with the level of ALT and AST but negative correlation with the level of dodecanoic acid (*r* = −0.25, *P* = 0.033) (Table 1; Fig. 6A). Also, taurine (*r* = −0.23, *P* = 0.044) had a positive correlation with the level of AST/ALT (Table 1; Fig. 6A).

**TABLE 1:** The correlation between selected metabolites with NAFLD indices^*a*^

NAFLD indices	Metabolites	Discovery population		Validation population	
		*r*	*P*	*r*	*P*
Gut microbiota					
*Alistipes*	Creatine	−0.23	0.047	−0.26	0.002
*Alistipes*	Creatinine	−0.23	0.044	−0.21	0.010
*Butyricicoccus*	Dodecanoic acid	−0.32	0.005	−0.22	0.008
*Alistipes*	Gallic acid	0.26	0.025	0.21	0.012
*Alistipes*	Taurine	−0.27	0.021	−0.34	<0.001
Inflammation					
IL-21	Dodecanoic acid	0.32	0.005	0.26	0.002
TNF-α	Dodecanoic acid	0.30	0.010	0.23	0.005
IL-1β	Dodecanoic acid	0.26	0.026	0.31	<0.001
Glycolipid metabolism					
TG	Creatinine	0.28	0.016	0.17	0.047
					
Liver function					
ALT	Dodecanoic acid	0.32	0.005	0.32	<0.001
AST/ALT	Dodecanoic acid	−0.25	0.033	−0.36	<0.001
AST	Dodecanoic acid	0.23	0.045	0.25	0.002
ALT	Taurine	0.37	0.001	0.24	0.004
AST	Taurine	0.33	0.003	0.21	0.013
AST/ALT	Taurine	−0.23	0.044	−0.24	*0.004*

^*a*^ AST, aspartate aminotransferase; ALT, alanine aminotransferase; TG, triglyceride; NAFLD, non-alcoholic fatty liver disease.

Similarly, the selected significant metabolites were compared with the gut microbiota and biochemical parameters in the validation population. Thus, when compared with the gut microbiota, *Alistipes* had a positive correlation with the gallic acid (*r* = 0.21, *P* = 0.012) but a negative correlation with the creatine (*r* = −0.26, *P* = 0.002), creatinine (*r* = −0.21, *P* = 0.010), and taurine (*r* = −0.34, *P* < 0.001) (Table 1; Fig. 6B). Moreover, *Butyricicoccus* had a negative correlation with the dodecanoic acid (*r* = −0.22, *P* = 0.008) (Table 1; Fig. 6B). A comparison with the inflammatory factors found that dodecanoic acid had a positive correlation with the IL-1β (*r* = 0.31, *P* < 0.001), IL-21 (*r* = 0.26, *P* = 0.002), and TNF-α (*r* = 0.23, *P* = 0.005) (Table 1; Fig. 6B). Furthermore, a comparison with the glycolipid metabolism found that creatinine (*r* = 0.17, *P* = 0.047) had a positive correlation with the level of TG (Table 1; Fig. 6B). Also, a comparison with the biochemical parameters of liver function found that dodecanoic acid (*r* = 0.32, *P* < 0.001 and *r* = 0.25, *P* = 0.002) and taurine (*r* = 0.24, *P* = 0.004 and *r* = 0.21, *P* = 0.013) had a positive correlation with the level of ALT and AST but a negative correlation with the level of dodecanoic acid (*r* = −0.36, *P* < 0.001). In addition, taurine (*r* = −0.24, *P* = 0.004) had a positive correlation with the level of AST/ALT (Table 1; Fig. 6B).

In order to validate the role of the metabolites of interest in NAFLD development, we explored the level of NAFLD indices in THLE-3 cells after the dodecanoic acid, creatinine, and taurine stimulation. Thus, when the THLE-3 cells were treated with 0.1 mM dodecanoic acid for 24 hours, the level of ALT (6.29 ± 0.94 U/gprot) was increased unlike in the control group. On the other hand, there was no significant difference in the levels of TNF-α between the treatment group and control group. Moreover, when the THLE-3 cells were treated with 0.25 mM dodecanoic acid for 24 hours, the levels of AST (46.55 ± 2.42 U/gprot) and IL-1β (7.04 ± 0.87 pg/mL) were more increased than those of the 0.1 mM group. Also, when the THLE-3 cells were treated with 0.5 mM creatinine, the level of TG (0.51 ± 0.06 mmol/gprot) was significantly more increased than that of the control group. Nevertheless, there was no significant difference in the levels of ALT and AST between the taurine treatment group and the control group (Fig. 6C).

## DISCUSSION

To the best of our knowledge, this is the first machine learning-based untargeted and targeted metabolomics to explore the relationship between NAFLD status and gut metabolomics. Untargeted metabolomics revealed a complete metabolic map of the gut, and RF machine learning and targeted metabolomics further identified novel gut metabolites (dodecanoic acid and creatinine) that could distinguish obese children from NAFLD children, especially NASH. Eventually, this study annotated novel elevated dodecanoic acid, creatinine, and the relationship between depleted protective gut microbiota (*Butyricicoccus* and *Alistipes*), inflammatory factors (IL-1β), disruption of lipid metabolism indicator (TG), and liver function indicators (ALT and AST). Pathway enrichment analyses showed that metabolites belonged to a major pathway, namely, biosynthesis of unsaturated fatty acids. The altered gut metabolites and their enriched functional pathways contributed at least in part to the NAFLD development, systemic inflammation, disruption of lipid metabolism, and liver function.

Previous studies have shown that diet plays an important role in metabolic diseases and the shaping of the gut microbiota (20, 21). In a longitudinal study of the gut microbiota, in infancy through early childhood, researchers tracked changes in diversity and composition associated with the development of the gut microbiota and associations with birth mode, diet, and atopic disorders (22). This study underscores the need for adjustment for diet as a confounding factor in the association between microbiota and disease outcomes. Therefore, we selected obese children as the control group and found no significant difference in diet between the case group and the control group. The possible reason for this outcome is that both the cases and controls were obese children. We speculate a small heterogeneity between obese children with regard to diet; hence, they were all exposed to unhealthy diets that can induce obesity. Thus, the purpose of controlling for the confounding factor of diet was achieved. Therefore, other factors may be influencing change in the gut microbiota, and accordingly, this study revealed associations between gut microbiota and its metabolites and NAFLD, after controlling for the confounding factor of diet.

Our research results showed that there was a negative correlation between *Butyricicoccus* and dodecanoic acid. Butyrate-producing gut bacteria, particularly *Butyricicoccus*, were known to be depleted in patients with metabolic syndrome, type 2 diabetes mellitus, and inflammatory bowel disease (23–25). Butyrate-producing gut bacteria and its metabolites are the major oxidative substrate for colonocytes (25, 26). More specifically, a previous study already pointed out that butyrate influences diverse cellular functions such as the immune function of intestinal epithelial cells and epithelial barrier function (27). Therefore, we speculate that the decrease in the abundance of *Butyricicoccus* may lead to decreased epithelial barrier integrity, further leading to the entry of harmful metabolites into the circulation, such as dodecanoic acid. Moreover, whether *Butyricicoccus* can inhibit the synthesis of dodecanoic acid needs further verification by additional in-depth studies, such as isolation and culture on bacterial experiment.

Our research results showed that there was a negative correlation between *Alistipes* and creatinine. Previous studies have found that the *Alistipes* were depleted in patients with obesity, type 2 diabetes mellitus, and inflammation liver cirrhosis (28–30). Fecal microbiome-based culture studies showed that *Alistipes* can hydrolyze tryptophan to indole and can produce succinic and acetic acid (31). Previous studies have suggested that indole and acetic acid have anti-inflammatory mechanisms; hence, it can be argued that the decrease in *Alistipes* can contribute to the decrease in indole and acetic acid and therefore can contribute to the NASH as observed in the NAFLD children. A study on mice with hepatocellular carcinoma receiving probiotics showed that probiotics shifted the gut microbiota community toward potential anti-inflammatory bacteria, including *Alistipes* (32). Moreover, it subsequently reduced the Th17 polarization and promoted the differentiation of Treg/Tr1 cells, an anti-inflammatory cell subset in the gut. It is noteworthy that whether *Alistipes* can reduce the production of pro-inflammatory metabolites such as creatinine needs further study.

Creatine belongs to the class of alpha amino acids and derivatives, mainly generated through the fermentation of fish, meat, and other animal products by gut microbiota in the bowel (33). The elevated creatinine concentration in the NASH group may be a metabolite of high concentration of creatine, generally known as pharmacologically inactive waste product (34). Nevertheless, creatine is commonly used as a dietary supplement by athletes to speed up recovery following strenuous training and enhances athletic performance (35, 36). It is important to note that most of the research has focused on elite athletes but relatively few studies have looked at the effects of creatine on the elderly or children (35, 37, 38). Some studies suggested that creatine was associated with the regulation of immune function, although other studies yielded contradictory results (39). In spite of that, creatine has been observed in several studies to downregulate cytokine expression (TNF-α, IL-1β, and IL-6) and repair impaired intestinal barrier function to reduce inflammation (40, 41). Moreover, although a recent study found that creatine ethyl ester (a creatine supplement) appeared to possess immunostimulatory properties and increased expression of pattern recognition receptors (TLR2, TLR3, TLR4, and TLR7) (42), other studies found contradictory results. Thus, our results showed that creatine and creatinine were negatively correlated with *Alistipes*, whereas creatinine was positively correlated with lipid metabolism index TG. Therefore, it is speculated that creatinine can promote the accumulation of TG through the biosynthesis pathway of unsaturated fatty acids.

Dodecanoic acid belongs to the class of medium-chain fatty acids. A previous study showed that dodecanoic acid plays an important role in the synthesis of LPS by bacteria (43). Specifically, the hydroxy fatty acid components of LPS at position 2′ are acylated at the 3-hydroxyl groups by dodecanoic acid in *Escherichia coli*. This is consistent with our findings. Note that LPS is a complex of lipids and polysaccharides mainly generated through the membrane component of Gram-negative bacteria. LPS has been observed to be strongly involved in the pathogenesis of NAFLD by triggering chronic low-grade inflammation (44). Consistently, our study selected dodecanoic acid as the key component in the NAFL-NASH machine learning models. The results showed that dodecanoic acid was negatively correlated with *Butyricicoccus* but positively correlated with liver function indices (ALT and AST) and inflammatory factors (IL-21, TNF-α, and IL-1β). Therefore, we hypothesize that dodecanoic acid is pro-inflammatory through the LPS biosynthetic pathway.

Previous studies showed that NAFLD patients and animals had significantly higher levels of LPS than healthy controls (44, 45). LPS also seems to promote IR, dyslipidemia, hepatic inflammation, and fibrosis through activation of Toll-like receptor 4 (TLR4) and LPS binding protein in the gut, liver, and adipose tissue (45, 46). Further activation of the Myosin light chain kinase , nuclear factor-κB (NF-κB), and IL-1R-associated kinase 4 was associated with impaired gut barrier function and enhanced transcription of pro-inflammatory mediators (47, 48). Additionally, these signalings were activated upon hepatic steatosis and liver damage recognition that induced a signaling cascade, resulting in the production of chemokines and pro-inflammatory cytokines, leading to aggravated disorder of hepatic inflammatory microenvironment, steatohepatitis, and fibrosis (47, 49).

Gallic acid belongs to the class of trihydroxybenzoic acid, widely isolated from a variety of herbs, plants, and fruits (50). This is in agreement with our findings. *Alistipes* had a positive correlation with the gallic acid. The anti-inflammatory properties and anti-oxidative role of gallic acid have been observed in numerous previous studies (51, 52). Importantly, there have been numerous studies indicating that gallic acid blocks the expression of the inflammatory mediators (IL-1β and TNF-α) evoked by NF-κB and downregulates pro-inflammatory signaling (nitrite, NO, PGE2, and IL-6) in a dose-dependent manner as well as inflicts oxidative damage in the liver (52, 53).

Taurine belongs to the class of amino sulfonic acid, abundantly expressed in red meat and organ meats (54). Indeed, compelling data have shown that taurine is an effective anti-inflammatory and anti-oxidant (55, 56). Our results showed that taurine was negatively correlated with *Alistipes* and positively correlated with liver function indices (ALT and AST). This result may suggest that high concentrations of taurine may be accompanied by high intake of red meats, which is an important risk factor for NAFLD and inflammation (57). Therefore, it is speculated that taurine has pro-inflammatory effect through pathways of taurine and hypotaurine metabolism. Collectively, these data suggest that gut metabolic alterations are different between children with NAFLD and the controls. We propose a hypothesis that unhealthy gut microbiota could induce the disorder of gut metabolites and these physiologically active metabolites further affect the occurrence and development of NAFLD, including systemic inflammation and disruption of glucose and lipid metabolism and liver function.

This study has illustrated a complete metabolic map of the gut metabolome characteristics of children with NAFLD. Multiple validation approaches (machine learning and targeted metabolomic) further refined the selection of novel gut metabolites in the validation cohort that could distinguish NAFLD children from the controls. These metabolites were potential novel biomarkers associated with impaired microbiota, liver function, and inflammation and were validated by experiments of hepatocyte cell lines. A few limitations of this study should be acknowledged. First, factors that can influence the gut microbiota and metabolites, such as ethnicity, were not evaluated. Without an external ethnicity validation data set beside the Han ethnicity, we are not sure if the results could be generalized to other ethnic groups. Second, because of the poor accessibility of liver biopsy, our study was limited by the use of gut data that may not reflect changes in the liver metabolites. Therefore, studies of liver metabolites are needed to identify the relationship between metabolic changes and NAFLD.

In summary, this study annotated novel elevated pathogenic metabolites (dodecanoic acid and creatinine) and the relationship between depleted protective gut microbiota (*Butyricicoccus* and *Alistipes*), inflammation (IL-1β), disruption of lipid metabolism (TG), and liver function (ALT and AST). These results suggest that dodecanoic acid and creatinine may serve as novel biomarkers for risk stratification in children with NAFLD, especially NASH. These may further affect inflammation, glucose and lipid metabolism disorders, and liver function in the pathogenesis of NAFLD in children through the key metabolic pathway of fatty acid biosynthesis.

## MATERIALS AND METHODS

### Human subjects

The Hospital Ethics Research Committee approved the study (XYGW201804). Parents or guardians of the enrolled children provided written informed consent and written assent. Patient recruitment and exclusion criteria are shown in the Supplementary material in Appendix 11. A flow chart of the patient recruitment and sampling of the study is shown in Fig. 1. A total of 220 children were consecutively recruited at the Institute of Child Health, Hunan Children’s Hospital (Changsha, China), and the fecal samples and blood samples were taken in duplicate. In the discovery population, the investigation was performed in fecal samples from 75 subjects (25 NAFL patients, 25 NASH patients, and 25 obese controls) between June and December 2019. In the independent validation population, the investigation was performed in fecal samples from 145 subjects (53 NAFL patients, 39 NASH patients, and 53 obese controls) between January 2020 and September 2021. Fresh fecal samples were obtained for gut microbiota and metabolites. The dietary data of children were investigated with a weekly food frequency scale, including cereals/potatoes, fish/poultry/meat/eggs, vegetables/fruits, and milk/beans. Anthropometric and demographic data are shown in the Supplementary material in Appendix 12.

### Analysis of gut metabolites by untargeted metabolomics

Fecal samples (100 mg) were ground with liquid nitrogen prior to analysis. The homogenate was resuspended using 0.1% formic acid in 80% methanol/water (wt/wt) and prechilled using 80% methanol (400 µL). The homogenate was centrifuged at 15,000 rpm and 4°C for 5 minutes after being incubated on ice for 5 minutes and was then diluted to a final concentration containing 53% methanol. Next, the supernatant (100 µL) was injected into the UHPLC-MS/MS system analysis after being centrifuged at 15,000 *g* and 4°C for 10 minutes. Each supernatant was divided into two fractions. One was for analysis using UPLC-MS/MS methods with positive electrospray ionization mode, and the eluents for the positive electrospray ionization mode were eluent A (0.1% FA in Water) and eluent B (methanol). The other one was for analysis using UPLC-MS/MS with the negative electrospray ionization mode, and the eluents for the negative electrospray ionization mode were eluent A (5 mM ammonium acetate, pH 9.0) and eluent B (methanol). A Hypesil Gold column (100 × 2.1 mm, 1.9 µm, Thermo Fisher, Germany) was used in UPLC. The mobile solvent gradient was set as follows: 2% B for 1.5 minutes, 2%–100% B for 12.0 minutes, 100% B for 14.0 minutes, 100%–2% B for 14.1 minutes, and 2% B for 17 minutes. Furthermore, the gradient elution for methods using Hypesil Gold column was performed in a 17-minute run when the polar mobile phase was gradually increased from 2% to 100%. The MS capillary temperature was 320°C, sheath gas flow rate was at 40 arb, aux gas flow rate was at 10 arb, and spray voltage was at 3.2 kV for both positive/negative electrospray ionization modes.

Compound Discoverer 3.1 (CD3.1, Thermo Fisher) was used for peak comparison, peak extraction, and missing value filling on each intestinal metabolite. These metabolites must meet the following criteria: actual mass tolerance of 5 ppm, retention time tolerance of 0.2 minutes, signal/noise ratio of 3, signal intensity tolerance of 30%, and minimum intensity of 100,000. Furthermore, peaks were matched with the mzCloud, mzVault, and MassList (Novogene Bioinformatics Institute, Beijing, China) databases to obtain accurate qualitative results. Data were normalized using the QC sample in equation (1) as follows: *R*_*i*_, relative quantitative value of sample metabolite; *R*_*r*_, raw relative quantitative value of sample metabolite; *S*_*r*_, sum of relative quantitative value of metabolite; and *S*_*q*_, sum of relative quantitative value of QC sample.


(1) $$R_{i}=\frac{R_{r}}{\left(S_{r}/S_{q}\right)}$$

The specific level of the annotated metabolite depends on which database matches the metabolite. According to metabolomics standards initiative, the IDC grade of metabolite annotated by mzCloud or mzVault is level 2 and the MassList is level 3. If there are matching results in the same two databases, the matching results of the database with the highest priority will be reserved according to the database priority, mzCloud > mzVault > MassList. In this study, the score values matched in the mzCloud and mzVault databases were selected, and the compounds with the highest scores were selected as detected results.

### Analysis of gut metabolites by targeted metabolomics

In the independent validation population, we selected 20 metabolites found by untargeted metabolomics in the discovery study. To extract metabolites from the samples, 800 µL of cold methanol/acetonitrile/water (2:2:1) extraction solvent was added to 100 mg sample and adequately vortexed. For absolute quantification of the metabolites, stock solutions of stable- isotope internal standards were added to the extraction solvent simultaneously. Then, the samples were under vigorous shaking for 2 minutes at 4°C and incubated on ice for 20 minutes and then centrifuged at 14,000 *g* for 20 minutes at 4°C. The supernatant was collected and flowed through a 96-well protein precipitation plate, and then, the elution was collected and dried in a vacuum centrifuge at 4°C. For LC-MS analysis, the samples were re-dissolved in 100 µL acetonitrile/water (1:1) solvent and transferred to LC vials.

Analyses were performed using an UHPLC (1290 Infinity LC, Agilent Technologies, USA) coupled to a QTRAP MS (6500, Sciex, USA). The analytes were separated on hydrophilic interaction liquid chromatography (HILIC) (Waters UPLC BEH Amide column, 2.1 mm × 100 mm, 1.7 µm) and C18 columns (Waters UPLC BEH C18-2.1 × 100 mm, 1.7 µm). For HILIC separation, the column temperature was set at 35°C and the injection volume was 2 µL. Mobile phase A consisted of 100 mM ammonium acetate and 1.2% ammonium hydroxide in water, and mobile phase B comprised acetonitrile. A gradient (85% B at 0–1 minutes, 80% B at 3–4 minutes, 70% B at 6 minutes, 50% B at 10–12.5 minutes, and 85% B at 12.6–18 minutes) was then initiated at a flow rate of 300 µL/minute. For reverse phase liquid chromatography (RPLC) separation, the column temperature was set at 40°C, and the injection volume was 2 µL. Mobile phase A consisted of 50 mM ammonium formate and 0.4% formic acid in water, whereas mobile phase B consisted of methanol. A gradient (5% B at 0 minute, 60% B at 5 minutes, 100% B at 11–13 minutes, and 5% B at 13.1–16 minutes) was then initiated at a flow rate of 400 µL/minute. The sample was placed at 4°C during the whole analysis process. The 6500 QTRAP (AB SCIEX, USA) was performed in positive and negative switch modes. The positive electrospray ionization mode source conditions were set as follows: source temperature: 550°C; ion Source Gas1 (Gas1): 55; Ion Source Gas2 (Gas2): 55; Curtain gas (CUR): 40; and ion Sapary Voltage Floating (ISVF): +4500 V. The negative electrospray ionization mode source conditions were set as follows: source temperature: 550°C; Gas1: 55; Gas2: 55; CUR: 40; and ISVF: −4500 V. In addition, the multiple reaction monitoring (MRM) method was used for mass spectrometry quantitative data acquisition.

MultiQuant (version 3.0) was used for quantitative data processing. The MultiQuant based tool for task-oriented data evaluation was established, which allows for extracting selected chromatographic peak information from the measured MRM data sets. MultiQuant Software can sort MRM data by quality or signal/noise ratio. An overlay view of multiple MRM transitions for the same species was used to assess the peak shape in order to scan for possible peak interferences in selected channels. The ratio of the peak area (area of the peak for substance/area of the peak of the internal standard) was used for obtaining the absolute quantitation for each substance according to the calibration curve. The QC samples were processed together with the biological samples. Metabolites in QC samples with a coefficient of variation of less than 30% were denoted as reproducible measurements. The data were normalized using QC samples as follows: raw quantitative value of sample metabolite/(sum of quantitative value of metabolite/sum of quantitative value of QC sample).

### Analysis of gut microbiota by 16S ribosomal RNA sequencing

Fecal samples were collected when the time demographic measurements were taken on the subjects. Immediately after collection, fecal samples were frozen at −20°C and transported (packed with dry ice) to the laboratory, where they were kept at a temperature of −80°C. Novogene Bioinformatics Institute (Beijing, China) performed the DNA extraction, sequencing, and analysis of the gut microbiomes. Genomic DNA of gut microbiota was isolated from fecal samples using the TIANGEN DNA isolation kit (Novogene, China) and mechanical lysis, according to the manufacturer’s guidelines.

The 16S rRNA gene, comprising V4 regions, was amplified using the primer pair F515 and R806 (5′-GTGCCAGCMGCCGCGGTAA-3′ and 5′-GGACTACVSGGGTATCTAAT-3′, respectively). Moreover, 16S rRNA genes of distinct regions were amplified using a specific bar-coded primer (16S V4: 515F-806R). All PCR reactions were carried out with 15 µL of Phusion High-Fidelity PCR Master Mix (New England Biolabs), 0.2 µM of forward and reverse primers, and about 10 ng template DNA. Thermal cycling consisted of initial denaturation at 98°C for 1 minute, followed by 30 cycles of denaturation at 98°C for 10 seconds, annealing at 50°C for 30 seconds, and elongation at 72°C for 30 seconds and, finally, 72°C for 5 minutes. The amplicons from the original DNA fragments were purified, quantified, and pooled at an equimolar ratio based on the method described previously. The same volume of 1× loading buffer (contained SYB green) was mixed with PCR products and operate electrophoresis on 2% agarose gel for detection. Also, PCR products were mixed in equidensity ratios. Then, mixtures of PCR products were purified with a Qiagen Gel Extraction Kit (Qiagen, Germany). Sequencing libraries were generated using a TruSeq DNA PCR-Free Sample Preparation Kit (Illumina, USA) following the manufacturer’s recommendations, and index codes were added. The library quality was assessed on the Qubit@2.0 Fluorometer (Thermo Scientific) and Agilent Bioanalyzer 2100 system. At last, the sequencing of 16S rRNA was performed on the Illumina MiSeq platform at the Novogene (NovaSeq6000, Beijing, China) of the V4 region (insert size 300 bp, read length 250 bp).

For taxonomic assignment, sequence reads were grouped into OTUs at a sequence similarity level of 97%. The raw sequences were first quality controlled using FLASH (version1.2.7) and QIIME (version 1.9) with default parameters (including dereplication, chimera filtering, and read error correction) and then grouped into OTUs using Uparse pipeline (v7.0.1001) at a sequence similarity level of 97%. For each representative sequence, the Silva database was used based on the Mothur algorithm to annotate taxonomic information. OTU abundance information was normalized using a standard of sequence number corresponding to the sample with the least sequences.

### Clinical laboratory data and inflammatory factors measurements

Fasting venous blood samples were collected from the veins of the subjects using a potassium EDTA tube after 12 hours of fasting. In addition, blood collection for measuring biochemical markers was performed. Thus, lipid profile and levels of biochemical markers, such as glucose, low-density cholesterol, high-density cholesterol, triglyceride, cholesterol, uric acid, insulin, serum C-peptide, hemoglobin, glycosylated hemoglobin, total bilirubin, direct bilirubin, indirect bilirubin, total protein, albumin, globulin, aspartate aminotransferase, and alanine aminotransferase, were measured using an autoanalyzer (OLYMPUS AU5400, Japan). The plasma levels of IL-1β, IL-6, IL-12, IL-17, IL-21, IL-32, TNF-α, and CXCL8 were measured using enzyme linked immunosorbent assay (ELISA) kits (Renjiebio, China) by Tecan Enzyme tag analyzer (Infinite F50, Switzerland).

### Cell lines

Human normal hepatocyte cell line hepatocytes (THLE-3) were obtained from American Type Culture Collection (Manassas, Virginia, USA), which were non-tumorigenic and negative for alpha fetoprotein expression. The cells were cultured in RPMI-1640 medium (GE Health Life Sciences, USA) supplemented with 10% (vol/ vol) fetal bovine serum (Gibco, USA), and 1% penicillin/streptomycin (100 IU/mL penicillin and 100 mg/mL streptomycin) (Gibco, USA) at 37°C in a humidified atmosphere of 5% CO_2_ (Thermo Fisher Scientific, USA). We terminated with 0.25% trypsin-EDTA (Gibco, USA) and collected logarithmic growing cells. One million THLE-3 cells were seeded in each well of six-well culture plates. Then, cells were exposed to medium with or without indicated metabolites supplemented for 24 hours.

### Cell glycolipid metabolism, liver function, and inflammation assay

THLE-3 cells were treated with the creatinine (0 mM, 0.01 mM, 0.1 mM, and 0.5 mM), taurine (0 mM, 0.01 mM, 0.1 mM, and 0.25 mM), and dodecanoic acid (0 mM, 0.01 mM, 0.1 mM, and 0.25 mM) for 24 hours, respectively. Cell homogenate glycolipid metabolism and liver function indicators including TG, ALT, and AST were measured using the enzymatic reagent kits (Jiancheng, Nanjing, China) according to the manufacturer’s instructions. The TNF-α, IL-1β, and IL-21 levels were measured using human ELISA kits (Renjiebio, China) by Tecan Enzyme tag analyzer (Infinite F50, Switzerland). All experiments were performed in three biological replicates and three technical replicates.

### Statistical analysis

The differences in anthropometric properties, demographic, and gut microbiota abundance data between the cases and the controls were explored using Student’s *t*-test, FC analysis, or Mann-Whitney U test (variables with two categories) and Kruskal-Wallis test (when variables had more than two categories) using SPSS (version 25.0). Exploratory analysis of the whole metabolomics data was performed applying PCA and OPLS-DA. The VIP score of each variable in the OPLS-DA model was calculated to indicate its contribution to the classification. Volcano plots were used to filter metabolites of interest (VIP value > 1 and *P* < 0.05).

Clustering heat maps and Spearman correlation were used to assess the correlation between numerical variables, and correlation plots were plotted by R packages, pheatmap, and corrplot. The functions of the metabolites and metabolic pathways were investigated using the KEGG database. In the machine learning RF analysis, 500 trees were built using R package RF with 10-fold cross-validation, and this was repeated 1,000 times. In the discovery population, important metabolites were selected with a mean decrease accuracy of top 30 using RF analysis. Furthermore, the results of RF analysis were combined with those of biological function annotation to select important metabolites for the next targeted metabolomics verification. The foregoing statistical methods were also applied for analyzing the validation population data. Unless otherwise stated, statistical tests were two-tailed at the 5% significance level.

## Data Availability

All the data are available upon request. The datasetsdata sets of gut microbiota are available in the NCBI Trace Archive NCBI Sequence Read Archive SRA database (PRJNA737039). Gut metabolite data to this article can be found online at MetaboLights database (PRJNA1032872).

## SUPPLEMENTAL MATERIAL

Supplemental material spectrum.05230-22-s0001.doc Supplemental tables, figures, and text.

ASM does not own the copyrights to Supplemental Material that may be linked to, or accessed through, an article. The authors have granted ASM a non-exclusive, world-wide license to publish the Supplemental Material files. Please contact the corresponding author directly for reuse.
