# Erratum for Luo et al., “Integrative metabolomics highlights gut microbiota metabolitesas novel NAFLD-related candidate biomarkers in children”

**DOI:** 10.1128/spectrum.01017-24

**Published:** 2024-07-01

**Authors:** Jiayou Luo, Miyang Luo, Atipatsa C. Kaminga, Jia Wei, Wen Dai, Yunlong Peng, Kunyan Zhao, Yamei Duan, Xiang Xiao, SiSi Ouyang, Zhenzhen Yao, Yixu Liu, Xiongfeng Pan

**Affiliations:** 1 Department of Maternal and Child Health, Xiangya School of Public Health, Central South University, Changsha, China; 2 Pediatrics Research Institute of Hunan Province, Hunan Children’s Hospital, The Affiliated Children’s Hospital of Xiangya School of Medicine, CentralSouth University, Changsha, China; 3 Department of Epidemiology and Health Statistics, Xiangya School of Public Health, Central South University, Changsha, China; 4 Saw Swee Hock School of Public Health, National University of Singapore, Singapore, Singapore; 5 Department of Mathematics and Statistics, Mzuzu University, Mzuzu, Malawi; 6 Department of Epidemiology and Health Statistics, Medical College of Soochow University, Suzhou, China; 7 School of Public Health, University of South China, Hengyang, China

## Erratum

Volume 12, no. 4, e05230-22, https://doi.org/10.1128/spectrum.05230-22. The author affiliations should appear as shown in this erratum.

Page 18, Acknowledgments: The last sentence should read “We also sincerely thank the distinguished editors and reviewers for their constructive comments that improved the clarity of this article.”

