# Supplementary material for: Integrative metabolomics highlights gut microbiota metabolites as novel NAFLD-related candidate biomarkers in children
Source: Microbiol Spectr. 2024 Mar 6;12(4):e05230-22. doi: 10.1128/spectrum.05230-22 (PMC10986516; doi:10.1128/spectrum.05230-22)
Supplement: Supplemental material — Supplemental tables, figures, and text. [file spectrum.05230-22-s0001.doc]

**Supplementary Information for: Integrative metabolomics highlights gut microbiota metabolites as novel NAFLD-related candidate biomarker in children**

Jiayou Luo^1,2^, Miyang Luo^2,3,4^, Atipatsa C. Kaminga^5^, Jia Wei^2^, Wen Dai^2^, Yunlong Peng^6^, Kunyan Zhao^7^, Yamei Duan^2^, Xiongfeng Pan^1,2*^

^1^Pediatrics Research Institute of Hunan Province, Hunan Children's Hospital, Changsha 410007, China

^2^Department of Maternal and Child Health, Xiangya School of Public Health, Central South University, Changsha, China

^3^Department of Epidemiology and Health Statistics, Xiangya School of Public Health, Central South University, Changsha, China

^4^Saw Swee Hock School of Public Health, National University of Singapore. Tahir Foundation Building, Singapore

^5^Department of Mathematics and Statistics, Mzuzu University, Mzuzu, Malawi

^6^Department of Epidemiology and Health Statistics, Medical College of Soochow University, Suzhou, China

^7^School of Public Health, University of South China, Hengyang, China

**^*^Correspondence to:**

Xiongfeng Pan PhD, Pediatrics Research Institute of Hunan Province, Hunan Children's Hospital, Changsha. 86 Ziyuan Rd, Changsha, Hunan, P.R. of China 410007. Tel: 86-152-00864658. Fax: 86-731-84805454. E-mail: pxfcsu@163.com

**Contents**

**Appendix 1: Clinical and standard laboratory data in different groups in validation population**

**Appendix 2: Mann-Whitney U rank sum test for inflammatory factors in different groups in discovery population**

**Appendix 3: Mann-Whitney U rank sum test for inflammatory factors in different groups in validation population**

**Appendix 4: χ^2^ test for dietary data in different groups in discovery population**

**Appendix 5: χ^2^ test for dietary data in different groups in validation population**

**Appendix 6: Pearson correlation between negative mode (A) and positive mode (B) quality control (QC) samples for untargeted metabolomics**

**Appendix 7: HMDB annotation plot of untargeted metabonomics**

**Appendix 8: Lipidmaps annotation plot of untargeted metabonomics**

**Appendix 9: Principal component analysis (PCA) of negative mode (A) and positive mode (B) metabolites**

**Appendix 10. List of metabolites included in the targeted LC-MS/MS analysis**

**Appendix 11. Human subjects**

**Appendix 12. Anthropometric and demographic measurements**

**Appendix 1: Clinical and standard laboratory data in different groups in** **validation population**

|  | NAFL | NASH | C | *F/H/χ^2^* | p |
| --- | --- | --- | --- | --- | --- |
| Age (years) | 11.00（10.00，12.00） | 11.00（10.00，12.00） | 11.00（9.50，12.00） | 0.65 | 0.724 |
| Gender |  |  |  | 0.70 | 0.748 |
| Boy | 40（75.5） | 32（82.1） | 40（75.5） | |  |
| Girl | 13（24.5） | 7（17.9） | 13（24.5） | |  |
| Body weight (kg) | 69.10（56.30，80.25） | 62.30（52.00，73.50） | 60.30（54.60，67.05） | 6.38 | 0.041 |
| Systolic blood pressure (mmHg) | 120.00（110.50，124.50） | 116.00（110.00，128.00） | 115.00（111.00，129.00） | 1.02 | 0.602 |
| Diastolic blood pressure (mmHg) | 69.00（65.25，73.50） | 75.00（66.00，78.00） | 70.00（66.00，76.00） | 5.68 | 0.058 |
| Intracellular water (kg) | 14.68（13.33，19.92） | 16.38（14.25，21.30） | 15.60（13.38，18.80） | 4.40 | 0.111 |
| Extracellular water (kg) | 9.17（8.19，12.02） | 10.24（8.90，13.12） | 10.00（8.21，11.65） | 4.28 | 0.118 |
| Protein (kg) | 7.97±1.87 | 7.41±1.81 | 7.19±1.32 | 3.03 | 0.052 |
| Inorganic salt (kg) | 2.76±0.62 | 2.62±0.68 | 2.50±0.54 | 2.47 | 0.088 |
| Body fat (kg) | 24.90（20.80，34.45） | 25.20（18.90，31.10） | 23.60（20.60，26.45） | 4.14 | 0.126 |
| Basal metabolism (kcal) | 1243.77±194.32 | 1184.93±198.86 | 1159.79±144.45 | 3.04 | 0.051 |
| BMI（kg/m^2^） | 27.80（25.80，31.85） | 27.40（25.20，29.90） | 27.70（26.50，28.55） | 1.31 | 0.52 |
| Total body moisture (kg) | 29.62±6.36 | 27.70±6.72 | 26.88±4.86 | 2.93 | 0.057 |
| Muscle mass (kg) | 26.48±8.99 | 25.46±10.71 | 31.33±8.86 | 5.41 | 0.005 |
| Fat free body weight (kg) | 40.76±8.82 | 37.83±9.21 | 36.35±6.83 | 3.89 | 0.023 |
| Visceral fat (cm^2^) | 135.20（131.25，135.21） | 152.2（144.90，152.20） | 110.10（87.52，131.75） | 13.72 | 0.001 |
| Body fat percentage (%) | 40.20（36.70，45.20） | 39.80（38.10，45.40） | 39.30（37.35，42.65） | 1.30 | 0.522 |
| Total bilirubin（μmol/L） | 9.40（7.75，12.20） | 11.20（8.30，15.20） | 9.20（7.00，11.70） | 5.23 | 0.073 |
| Direct bilirubin（μmol/L） | 2.50（2.20，3.30） | 2.90（2.40，3.80） | 2.60（1.75，3.43） | 3.92 | 0.141 |
| Indirect bilirubin（μmol/L） | 7.37±2.74 | 8.84±4.42 | 6.94±2.74 | 3.98 | 0.021 |
| Total protein (g/L) | 71.11±3.91 | 71.35±3.32 | 71.98±4.52 | 0.65 | 0.521 |
| Albumin (g/L) | 42.11±2.26 | 42.35±2.47 | 43.19±3.23 | 2.27 | 0.107 |
| Globulin (g/L) | 29.19±2.91 | 29.00±2.71 | 28.79±2.98 | 0.25 | 0.778 |
| Alanine aminotransferase (IU/L) | 29.90（19.55，43.25） | 105.40（75.10，121.50） | 19.80（15.90，28.20） | 85.25 | <0.001 |
| Glutamic oxaloacetic transaminase (IU/L) | 25.20（21.00，29.70） | 53.90（47.50，63.00） | 21.65（17.50，27.65） | 79.63 | <0.001 |
| Total bile acid（μmol/L） | 2.30（1.60，3.80） | 3.40（2.30，6.10） | 2.30（1.50，3.30） | 7.30 | 0.026 |
| Urea nitrogen (mmol/L) | 3.63（3.13，4.45） | 3.93（3.31，4.65） | 4.14（3.34，4.58） | 3.15 | 0.207 |
| Creatinine（μmol/L） | 48.33±8.02 | 46.51±8.17 | 46.83±8.49 | 0.68 | 0.507 |
| Uric acid（μmol/L） | 433.66±99.20 | 419.18±89.81 | 388.97±91.72 | 3.09 | 0.049 |
| Triglycerides (mmol/L) | 1.46（1.08，2.03） | 1.69（1.11，2.13） | 1.21（0.76，1.52） | 14.29 | 0.001 |
| Cholesterol (mmol/L) | 4.14（3.71，4.54） | 4.05（3.53，4.72） | 3.82（3.31，4.33） | 5.03 | 0.081 |
| High density lipoprotein cholesterol (mmol/L) | 1.01（0.94，1.23） | 1.04（0.92，1.18） | 1.17（1.03，1.34） | 9.64 | 0.008 |
| Low density lipoprotein cholesterol (mmol/L) | 2.44（1.94，2.79） | 2.51（2.04，3.03） | 2.00（1.78，2.46） | 11.07 | 0.004 |
| Glucose (mmol/L) | 4.61（4.25，4.89） | 4.60（4.23，4.79） | 4.63（4.33，4.87） | 0.38 | 0.828 |
| Hemoglobin (g/dl) | 15.17（13.83，15.97） | 16.03（15.30，16.20） | 15.50（14.23，16.23） | 12.31 | <0.001 |
| Glycosylated hemoglobin (g/dl) | 0.78（0.73，0.84） | 0.83（0.78，0.85） | 0.72（0.70，0.77） | 32.74 | <0.001 |
| Glycated hemoglobin ratio (%) | 5.15（4.13，5.63） | 5.63（5.15，6.18） | 4.50（3.94，4.89） | 27.21 | <0.001 |
| Serum insulin（μU/mL） | 17.35（12.08，37.28） | 29.06（18.74，43.65） | 21.08（13.09，30.02） | 10.22 | 0.006 |
| Serum C peptide (ng/ml) | 2.87（2.04，4.40） | 3.52（2.66，5.14） | 2.95（2.26，3.82） | 8.79 | 0.012 |

NAFL, nonalcoholic fatty liver; NASH, non-alcoholic steatohepatitis.

**Appendix 2: Mann-Whitney U rank sum test for inflammatory factors** in different groups in discovery population

|  | **Control** | **NAFL** | **NASH** | **p for two groups** | |  |
| --- | --- | --- | --- | --- | --- | --- |
|  |  |  |  | **Control-NAFL** | **Control-NASH** | **NAFL-NASH** |
| **IL1B (pg/mL)** | 10.05 ±1.67 | 14.34 ±2.16 | 15.23 ±2.18 | <0.001 | <0.001 | 0.152 |
| **IL6 (pg/mL)** | 7.16 ±0.75 | 9.01 ±1.18 | 9.52 ±1.33 | <0.001 | <0.001 | 0.155 |
| **IL12 (pg/mL)** | 8.47 ±0.94 | 9.91 ±0.80 | 11.02 ±1.35 | <0.001 | <0.001 | 0.001 |
| **IL17 (pg/mL)** | 31.68 ±6.50 | 53.15 ±6.98 | 54.27 ±6.62 | <0.001 | <0.001 | 0.564 |
| **IL21 (pg/mL)** | 45.63 ±7.12 | 65.17 ±6.99 | 63.33 ±8.14 | <0.001 | <0.001 | 0.395 |
| **IL32 (pg/mL)** | 19.21 ±2.12 | 22.93 ±3.28 | 24.35 ±3.69 | <0.001 | <0.001 | 0.133 |
| **TNFa (pg/mL)** | 10.28 ±1.59 | 13.35 ±2.24 | 14.79 ±2.32 | <0.001 | <0.001 | 0.030 |
| **CXCL8 (pg/mL)** | 1.08 ±0.22 | 1.52 ±0.29 | 1.68 ±0.28 | <0.001 | <0.001 | 0.043 |

NAFL, nonalcoholic fatty liver; NASH, non-alcoholic steatohepatitis.

**Appendix 3: Mann-Whitney U rank sum test for inflammatory factors** in different groups in validation population

|  |  |  |  | **p for two groups** | |  |
| --- | --- | --- | --- | --- | --- | --- |
|  | **Control** | **NAFL** | **NASH** | **Control-NAFL** | **Control-NASH** | **NAFL-NASH** |
| **IL1B (pg/mL)** | 9.68 ±1.88 | 13.48 ±2.20 | 15.14 ±2.06 | <0.001 | <0.001 | <0.001 |
| **IL6 (pg/mL)** | 6.65 ±1.14 | 8.68 ±1.12 | 9.71 ±1.06 | <0.001 | <0.001 | <0.001 |
| **IL12 (pg/mL)** | 7.87 ±1.34 | 10.33 ±1.38 | 10.39 ±1.54 | <0.001 | <0.001 | 0.937 |
| **IL17 (pg/mL)** | 32.51 ±6.36 | 47.66 ±8.84 | 57.49 ±8.17 | <0.001 | <0.001 | <0.001 |
| **IL21 (pg/mL)** | 44.50 ±6.38 | 60.15 ±7.74 | 59.97 ±8.54 | <0.001 | <0.001 | 0.893 |
| **IL32 (pg/mL)** | 17.09 ±3.37 | 22.73 ±2.93 | 22.58 ±3.14 | <0.001 | <0.001 | 0.816 |
| **TNFa (pg/mL)** | 10.07 ±1.43 | 13.34 ±1.77 | 14.80 ±2.25 | <0.001 | <0.001 | 0.001 |
| **CXCL8 (pg/mL)** | 0.98 ±0.23 | 1.41 ±0.23 | 1.64 ±0.31 | <0.001 | <0.001 | <0.001 |

NAFL, nonalcoholic fatty liver; NASH, non-alcoholic steatohepatitis.

**Appendix 4: χ^2^ test for dietary data in different groups in discovery population**

| **Dietary frequency** | | **Control** | **NAFL** | **NASH** | **χ^2^** | **p** |
| --- | --- | --- | --- | --- | --- | --- |
| **Cereals/potatoes** | 4~6 | 2 | 0 | 0 | 2.737^ | 0.324 |
|  | 7 | 23 | 25 | 25 |  |  |
| **Fish/poultry/meat/eggs** | 0 | 0 | 0 | 1 | 6.704^ | 0.248 |
|  | 1~3 | 1 | 0 | 2 |  |  |
|  | 4~6 | 3 | 1 | 0 |  |  |
|  | 7 | 21 | 24 | 22 |  |  |
| **Vegetables/fruits** | 0 | 2 | 0 | 0 | 5.001^ | 0.545 |
|  | 1~3 | 5 | 4 | 5 |  |  |
|  | 4~6 | 4 | 2 | 5 |  |  |
|  | 7 | 14 | 19 | 15 |  |  |
| **Milk/beans** | 0 | 6 | 1 | 2 | 5.320^ | 0.512 |
|  | 1~3 | 7 | 7 | 7 |  |  |
|  | 4~6 | 3 | 6 | 5 |  |  |
|  | 7 | 9 | 11 | 11 |  |  |

NAFL, nonalcoholic fatty liver; NASH, non-alcoholic steatohepatitis, ^Fisher exact test.

**Appendix 5: χ^2^ test for dietary data in different groups in validation populatio**n

| **Dietary frequency** | | **Control** | **NAFL** | **NASH** | **χ^2^** | **p** |
| --- | --- | --- | --- | --- | --- | --- |
| **Cereals/potatoes** | 0 | 1 | 1 | 0 | 4.854^ | 0.858 |
|  | 1~3 | 0 | 0 | 1 |  |  |
|  | 4~6 | 1 | 0 | 0 |  |  |
|  | 7 | 51 | 52 | 38 |  |  |
| **Fish/poultry/meat/eggs** | 0 | 1 | 2 | 0 | 8.397^ | 0.136 |
|  | 1~3 | 1 | 1 | 3 |  |  |
|  | 4~6 | 7 | 1 | 3 |  |  |
|  | 7 | 44 | 49 | 33 |  |  |
| **Vegetables/fruits** | 0 | 2 | 1 | 1 | 5.871^ | 0.422 |
|  | 1~3 | 8 | 6 | 7 |  |  |
|  | 4~6 | 5 | 4 | 8 |  |  |
|  | 7 | 38 | 42 | 23 |  |  |
| **Milk/beans** | 0 | 5 | 4 | 2 | 5.723^ | 0.456 |
|  | 1~3 | 14 | 18 | 13 |  |  |
|  | 4~6 | 8 | 4 | 9 |  |  |
|  | 7 | 26 | 27 | 15 |  |  |

NAFL, nonalcoholic fatty liver; NASH, non-alcoholic steatohepatitis, ^Fisher exact test.

**Appendix 6: Pearson correlation between negative mode (A) and positive mode (B) quality control (QC) samples for** **untargeted metabolomics**

**
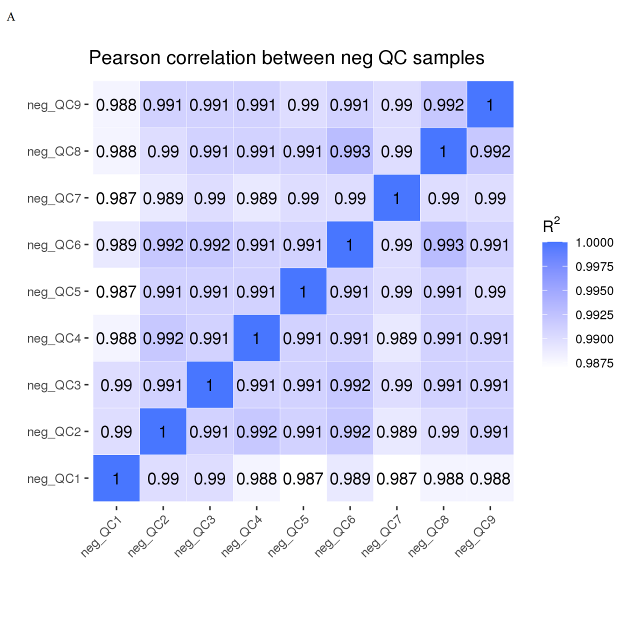
**

**
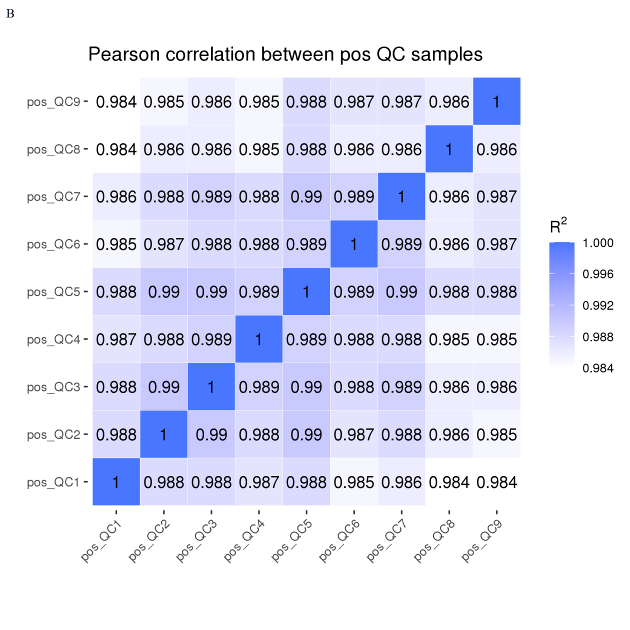
**

The higher the correlation of QC samples (the closer R^2^ is to 1), the better the stability of the whole detection process and the higher the data quality.

**Appendix 7: HMDB annotation plot of untargeted metabonomics**


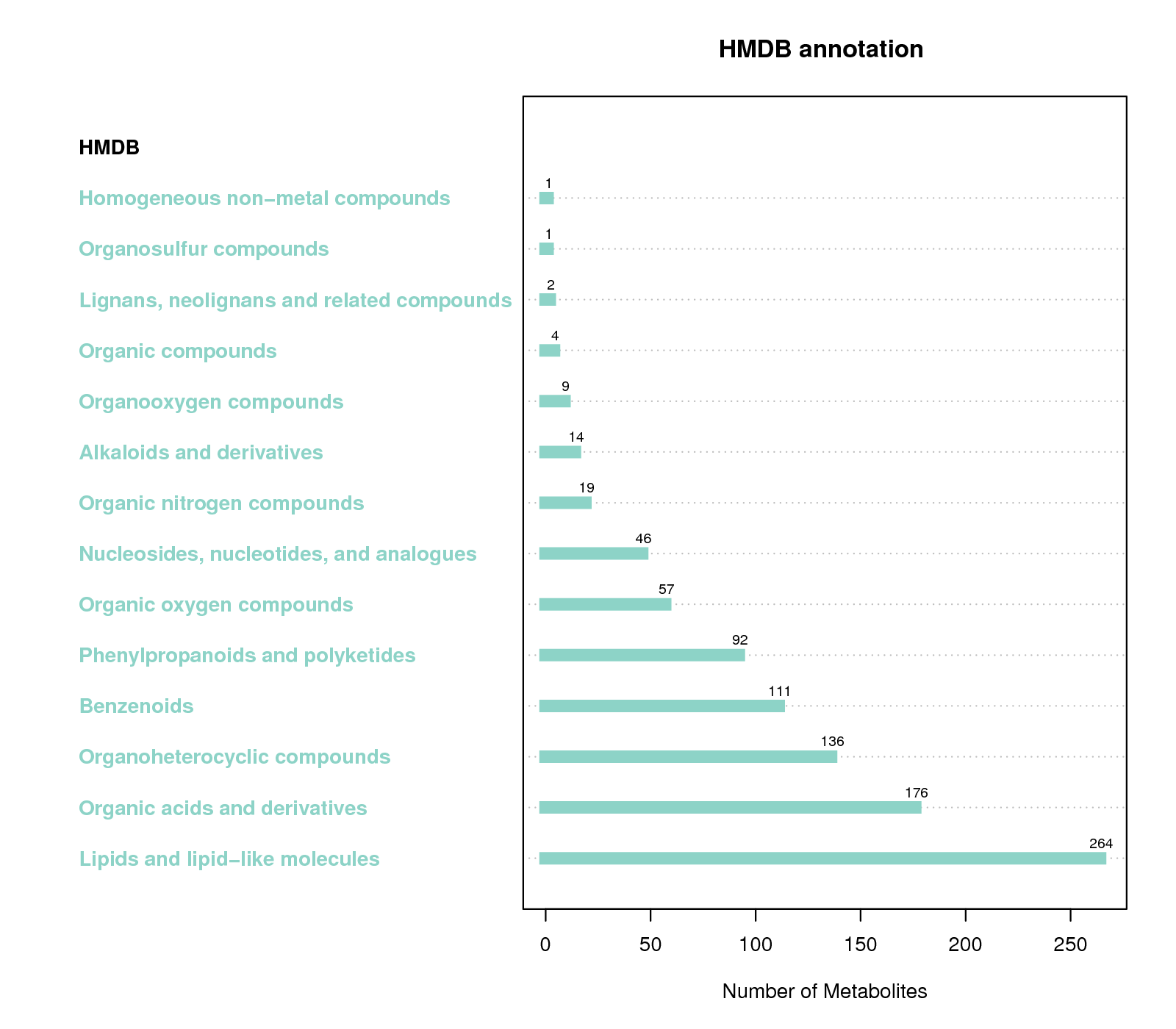


**Appendix 8: Lipidmaps annotation plot of untargeted metabonomics**


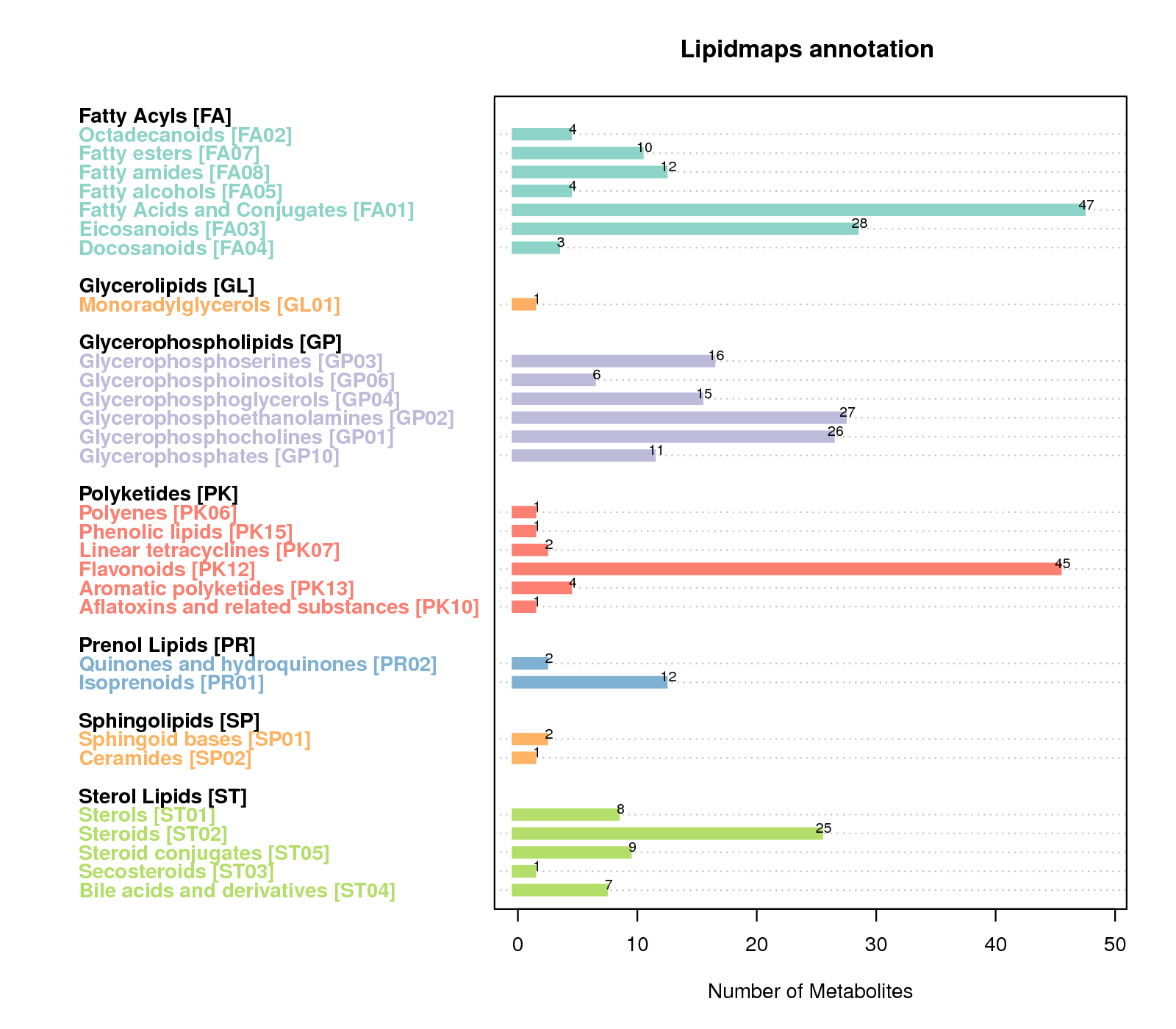


**Appendix 9: Principal component analysis (PCA) of negative mode (A) and positive mode (B) metabolites**


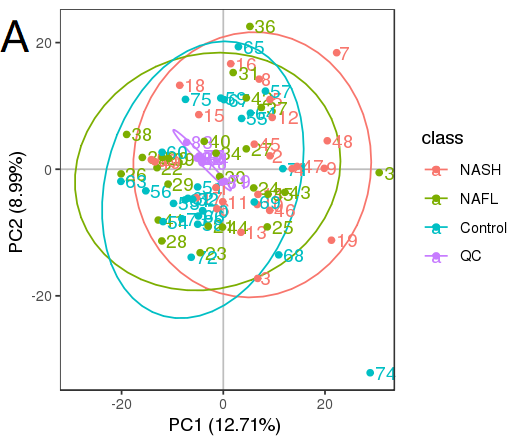

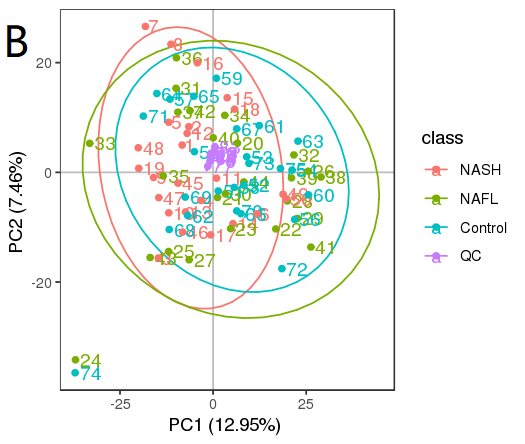


NAFL, nonalcoholic fatty liver; NASH, non-alcoholic steatohepatitis.

**Appendix 10**. List of metabolites included in the targeted LC-MS/MS analysis

| **Component Name** | **Class** | **HMDB ID** | **PubChem ID** | **RT** | **LOQ (ng/mL)** | **LOD (ng/mL)** | **Q1** | **Q3** | **DP** | **CE** |
| --- | --- | --- | --- | --- | --- | --- | --- | --- | --- | --- |
| Creatine | Amino acids | HMDB0000064 | 586 | 2.16 | 5 | 1 | 132.0 | 90.1 | 71 | 18 |
| 4-Hydroxyphenylpyruvic acid | Benzenoids | HMDB0000707 | 979 | 3.03 | 10 | 5 | 179.1 | 92.1 | -86 | -35 |
| Gallic acid | Benzenoids | HMDB0005807 | 370 | 0.86 | 20 | 5 | 169.0 | 125.0 | -98 | -20 |
| Salicyluric acid | Benzenoids | HMDB0000840 | 10253 | 3.57 | 1 | 0.5 | 194.0 | 93.0 | -71 | -31 |
| Cholic acid (CA) | Bile acids | HMDB0000619 | 221493 | 8.98 | 1 | 0.5 | 407.4 | 407.4 | -165 | -45 |
| Isolithocholic acid (IsoLCA) | Bile acids | HMDB0000717 | 9903 | 10.04 | 200 | 50 | 375.5 | 375.5 | -173 | -25 |
| 7Z,10Z,13Z,16Z-Docosatetraenoic acid | Fatty acids | HMDB0002226 | 5497181 | 11.69 | 100 | 50 | 331.3 | 331.3 | -171 | -5 |
| Arachidic Acid | Fatty acids | HMDB0002212 | 10467 | 12.58 | 200 | 50 | 311.3 | 311.3 | -115 | -5 |
| Arachidonic acid | Fatty acids | HMDB0001043 | 444899 | 11.26 | 5 | 1 | 303.2 | 303.2 | -115 | -5 |
| Dodecanoic acid | Fatty acids | HMDB0000638 | 3893 | 10.18 | 50 | 15 | 199.2 | 199.2 | -114 | -5 |
| Palmitic acid | Fatty acids | HMDB0000220 | 985 | 11.58 | 1000 | 300 | 255.2 | 255.2 | -114 | -5 |
| Pentadecanoic acid | Fatty acids | HMDB0000826 | 13849 | 3.60 | 50 | 15 | 241.2 | 241.2 | -114 | -5 |
| Stearic acid | Fatty acids | HMDB0000827 | 5281 | 12.08 | 1000 | 300 | 283.3 | 283.3 | -114 | -5 |
| Suberic acid | Fatty acids | HMDB0000893 | 10457 | 4.47 | 1 | 0.5 | 173.1 | 111.2 | -70 | -18 |
| Linoleoyl ethanolamide | Fatty acyl | HMDB0012252 | 5283446 | 10.76 | 50 | 15 | 324.3 | 62.1 | 166 | 18 |
| Serotonin | Indoles | HMDB0000259 | 5202 | 0.86 | 50 | 15 | 177.2 | 160.1 | 70 | 16 |
| Taurine | Organosulfonic acids and derivatives | HMDB0000251 | 1123 | 5.06 | 200 | 50 | 126.0 | 44.1 | 50 | 23 |
| 3-methoxytyramine | Phenols | HMDB0000022 | 1669 | 1.87 | 20 | 5 | 168.2 | 151.0 | 40 | 15 |
| Creatinine | Purine nucleotides | HMDB0000562 | 588 | 2.16 | 10 | 2 | 114.1 | 44.0 | 55 | 28 |
| Pyridoxal VB6 | Purine nucleotides | HMDB0001545 | 1050 | 1.74 | 200 | 50 | 168.0 | 150.1 | 56 | 30 |

**Appendix 11. Human subjects**

A total of 220 Children with 440 fecal samples and 440 blood samples were consecutively included at the Institute of Child Health, Hunan children's Hospital (Changsha, China). According to the standard of "Screening for overweight and obesity among school-age children and adolescents" (WS/T 586-2018), the BMI status of children were classified into 3 categories, normal, overweight and obesity (**Table S1**).

**Ta**ble S1. Screening for overweight and obesity among children and adolescents

| **Age** |  | **Boy** |  |  | **Girl** |  |
| --- | --- | --- | --- | --- | --- | --- |
|  | **Normal** | **Overweight** | **Obesity** | **Normal** | **Overweight** | **Obesity** |
| 6.0- | 13.4 | 16.4 | 17.7 | 13.1 | 16.2 | 17.5 |
| 6.5- | 13.8 | 16.7 | 18.1 | 13.3 | 16.5 | 18.0 |
| 7.0- | 13.9 | 17.0 | 18.7 | 13.4 | 16.8 | 18.5 |
| 7.5- | 13.9 | 17.4 | 19.2 | 13.5 | 17.2 | 19.0 |
| 8.0- | 14.0 | 17.8 | 19.7 | 13.6 | 17.6 | 19.4 |
| 8.5- | 14.0 | 18.1 | 20.3 | 13.7 | 18.1 | 19.9 |
| 9.0- | 14.1 | 18.5 | 20.8 | 13.8 | 18.5 | 20.4 |
| 9.5- | 14.2 | 18.9 | 21.4 | 13.9 | 19.0 | 21.0 |
| 10.0- | 14.4 | 19.2 | 21.9 | 14.0 | 19.5 | 21.5 |
| 10.5- | 14.6 | 19.6 | 22.5 | 14.1 | 20.0 | 22.1 |
| 11.0- | 14.9 | 19.9 | 23.0 | 14.3 | 20.5 | 22.7 |
| 11.5- | 15.1 | 20.3 | 23.6 | 14.5 | 21.1 | 23.3 |
| 12.0- | 15.4 | 20.7 | 24.1 | 14.7 | 21.5 | 23.9 |
| 12.5- | 15.6 | 21.0 | 24.7 | 14.9 | 21.9 | 24.5 |
| 13.0- | 15.9 | 21.4 | 25.2 | 15.3 | 22.2 | 25.0 |
| 13.5- | 16.1 | 21.9 | 25.7 | 15.6 | 22.6 | 25.6 |
| 14.0- | 16.4 | 22.3 | 26.1 | 16.0 | 22.8 | 25.9 |
| 14.5- | 16.7 | 22.6 | 26.4 | 16.3 | 23.0 | 26.3 |
| 15.0- | 16.9 | 22.9 | 26.6 | 16.6 | 23.2 | 26.6 |
| 15.5- | 17.0 | 23.1 | 26.9 | 16.8 | 23.4 | 26.9 |
| 16.0- | 17.3 | 23.3 | 27.1 | 17.0 | 23.6 | 27.1 |
| 16.5- | 17.5 | 23.5 | 27.4 | 17.1 | 23.7 | 27.4 |
| 17.0- | 17.7 | 23.7 | 27.6 | 17.2 | 23.8 | 27.6 |
| 17.5- | 17.9 | 23.8 | 27.8 | 17.3 | 23.9 | 27.8 |
| 18.0- | 17.9 | 24.0 | 28.0 | 17.3 | 24.0 | 28.0 |

In discovery population, the investigation was performed in fecal samples from 75 subjects (25 NAFL patients, 25 NASH patients, and 25 obese controls) between June and December 2019. In the independent validation population, the investigation was performed in fecal samples from 145 subjects (53 NAFL patients, 39 NASH patients, and 53 obese controls) from January 2020 to September 2021. The Hunan children's Hospital Ethics Research Committee approved the study. Enrolled children their parent or guardian provided written informed consent and written assent. Clinical assessment (height, weight, glucose, and lipid, etc.), demographic data for each child were obtained at Hunan children's Hospital. Fresh fecal samples were obtained for gut microbiota and metabolites.

**Patient recruitment and inclusion and exclusion criteria**

Eligible subjects fulfilled the following inclusion criteria: (1) obese children ranging in age from 7 to 16 years recruited from Institute of Child Health; (2) NAFLD group were obese children with NAFLD diagnosed within 90 days defined by ultrasound detected; (3) control group were obese children without evidence of NAFLD based on ultrasound detected and clinical history; (4) NAFLD group were further divided into NAFL and NASH by biopsy and alanine aminotransferase (ALT) levels > 60 IU/L. Subjects were excluded for the following exclusion criteria: (1) current or past history of viral hepatitis, autoimmune liver disease, gastrointestinal operations, inflammatory bowel disease or other liver diseases; (2) excessive alcohol consumption, the alcohol consumption >140g/week for boys and >70 g/week for girls; (3) bacterial infection, use of steatosis-inducing drugs or antibiotics within the 90 days before sampling.

**Appendix 12. Anthropometric and demographic measurements**

Ultrasongraphic evaluation, using an ultrasound multifrequency curvilinear 3.5 to 5 MHz probe, was performed on the enrolled children by two expert radiologists to detect the occurrence of fatty liver. Anthropometric measurements were performed by trained nurses using standard protocols and calibrated instruments. Weight and height of participants were measured with light clothes and without shoes. Blood pressure was measured using a sphygmomanometer for Children. BMI was calculated as weight (in kilograms) divided by square of height (in meters). Waist circumference (WC) was measured by using a non-elastic tape around midway between the lower border of the rib cage and the iliac crest at the end of normal expiration. Demographic data were collected using a questionnaire that was designed by the Delphi method. The dietary data of children were investigated with a weekly food frequency scale, including cereals/potatoes, fish/poultry/meat/eggs, vegetables/fruits, and milk/beans (Table S2).

**Table S2. Weekly food frequency scale**

| **Dietary data** | **Weekly food frequency scale** | | | |
| --- | --- | --- | --- | --- |
|  | **0** | **1~3** | **4~6** | **7** |
| Cereals/potatoes |  |  |  |  |
| Fish/poultry/meat/eggs |  |  |  |  |
| Vegetables/fruits |  |  |  |  |
| Milk/beans |  |  |  |  |
